# Self-accelerating H_2_O_2_-responsive Plasmonic Nanovesicles for Synergistic Chemo/starving therapy of Tumors

**DOI:** 10.7150/thno.45392

**Published:** 2020-07-09

**Authors:** Yao Tang, Yuejia Ji, Chenglin Yi, Di Cheng, Bin Wang, Yun Fu, Yufang Xu, Xuhong Qian, Yahya E. Choonara, Viness Pillay, Weiping Zhu, Yunen Liu, Zhihong Nie

**Affiliations:** 1 State Key Laboratory of Bioreactor Engineering, Shanghai Key Laboratory of Chemical Biology, School of Pharmacy, East China University of Science and Technology, Shanghai 200237, China.; 2 State Key Laboratory of Molecular Engineering of Polymers, Department of Macromolecular Science, Fudan University, Shanghai 200438, China.; 3 Department of Pharmacy and Pharmacology, University of the Witwatersrand, Parktown 2193 Johannesburg, South Africa.; 4 Department of Emergency Medicine, the General Hospital of Northern Theater Command, Laboratory of Rescue Center of Severe Trauma PLA, Shenyang l10016, China.

**Keywords:** hydrogen peroxide, gold vesicles, cancer therapy, controlled release, cancer imaging, self-accelerating

## Abstract

**Rationale:** Nanoscale vehicles responsive to abnormal variation in tumor environment are promising for use in targeted delivery of therapeutic drugs specifically to tumor sites. Herein, we report the design and fabrication of self-accelerating H_2_O_2_-responsive plasmonic gold nanovesicles (GVs) encapsulated with tirapazamine (TPZ) and glucose oxidase (GOx) for synergistic chemo/starving therapy of cancers.

**Methods:** Gold nanoparticles were modified with H_2_O_2_-responsive amphiphilic block copolymer PEG_45_-*b*-PABE_330_ by ligand exchange. The TPZ and GOx loaded GVs (TG-GVs) were prepared through the self-assembly of PEG_45_-*b*-PABE_330_ -grafted nanoparticles together with TPZ and GOx by solvent displacement method.

**Results:** In response to H_2_O_2_ in tumor, the TG-GVs dissociate to release the payloads that are, otherwise, retained inside the vesicles for days without noticeable leakage. The released GOx enzymes catalyze the oxidation of glucose by oxygen in the tumor tissue to enhance the degree of hypoxia that subsequently triggers the reduction of hypoxia-activated pro-drug TPZ into highly toxic free radicals. The H_2_O_2_ generated in the GOx-catalyzed reaction also accelerate the dissociation of vesicles and hence the release rate of the cargoes in tumors. The drug-loaded GVs exhibit superior tumor inhibition efficacy in 4T1 tumor-bearing mice owing to the synergistic effect of chemo/starvation therapy, in addition to their use as contrast agents for computed tomography imaging of tumors.

**Conclusion:** This nanoplatform may find application in managing tumors deeply trapped in viscera or other important tissues that are not compatible with external stimulus (e.g. light).

## Introduction

The fast proliferation of tumor cells continuously produces H_2_O_2_ in large quantities (~50-100 μM) due to mitochondrial dysfunction 1, 2. The high-level H_2_O_2_, in turn, activates the related signal pathways to accelerate the proliferation of tumor cells and their invasion toward health tissues 3. The design of delivery vehicles that can respond to endogenous H_2_O_2_ enables controlled release of therapeutic drugs specifically at the tumor sites, thus reducing the side effect arising from the nonspecific accumulation of toxic drug molecules in healthy tissues 4-8. Furthermore, the rapid proliferation of tumor cells avidly consumes nutrients (e.g., glucose) and oxygen, causing a hypoxia environment in tumors. The depletion of oxygen triggers the glycolysis process in the tumor cells to gain energy in an efficient manner, which further accelerates the deprivation of glucose in tumor. Suppressing the supply of glucose and/or oxygen can inhibit the growth of tumor cells, thus achieving effective starvation therapy 9-11. Despite tremendous efforts toward tumor therapy, mono-modality therapy often does not provide satisfactory therapeutic effect in managing cancer patients due to the high heterogeneity and pathological complexity of tumors. One promising strategy for maximizing the outcomes of cancer treatment is to integrate multiple types of therapeutic modality in a single platform 12, 13. For instance, nitric oxide-based gas therapy overcomes the drug resistance of irinotecan or other drugs in chemotherapy 14-17. Photodynamic therapy produces singlet oxygen to strengthen the efficacy of paclitaxel-based chemotherapy 18. Among others, the combination of starving therapy and chemotherapy is promising for realizing an optimal therapeutic effect due to the different mechanism of the two modalities 19, 20.

Nanovesicles (e.g., liposomes and polymersomes) are assembled from amphiphilic biological or synthetic molecules (e.g., lipids, block copolymers). They are featured with a hollow cavity for encapsulating hydrophilic compounds and vesicular membranes for loading hydrophobic cargoes simultaneously. In the past decades, nanovesicles have been intensively explored as vehicles for delivering imaging and therapeutic agents, owing to their high loading capacity, tailorable response to various stimuli and versatile functionalities 21. Hybridization of organic nanovesicles with inorganic nanoparticles (e.g., gold and superparamagnetic iron oxide nanoparticles) can impart the platforms with unique optical, electronic and magnetic properties originated from inorganic components 22-25 or synergistic properties due to the coupling interactions between nanoparticles 26-28. These collective properties make the hybrid nanovesicles attractive for use in imaging, diagnosis and therapy 29-34. In particular, plasmonic gold nanovesicles (GVs) with a monolayer of densely packed gold nanoparticles (GNPs) show characteristic features such as tunable absorption in the near-infrared range, light-triggered release of payload, etc 26, 29, 34, 35. They have been demonstrated for effective photo-based imaging (e.g., photothermal and photoacoustic imaging) and therapy (e.g., photothermal, photodynamic, and chemo-therapy) of tumors 26, 29, 35-38. However, external stimuli (e.g., light) are usually used to trigger the release of payload, which may not be always practical or convenient for use under conditions lack of relevant clinic settings or incompatible with the stimuli (e.g., limited penetration depth of light for tumors deeply trapped in viscera or other important tissues). The design of hybrid nanovesicles that are responsive to internal pathological features of tumors (such as hypoxia 39, high glutathione (GSH) 40-43, acid microenvironment 44-50, elevated level of reactive oxygen species (ROS) 51-55, high glucose 15 level etc.) offers a promising route to overcome the limitations of photo-based therapeutic strategies. Nevertheless, there has been no report on H_2_O_2_-responsive GVs and their use for chemo/starvation therapy.

**Scheme 1:**
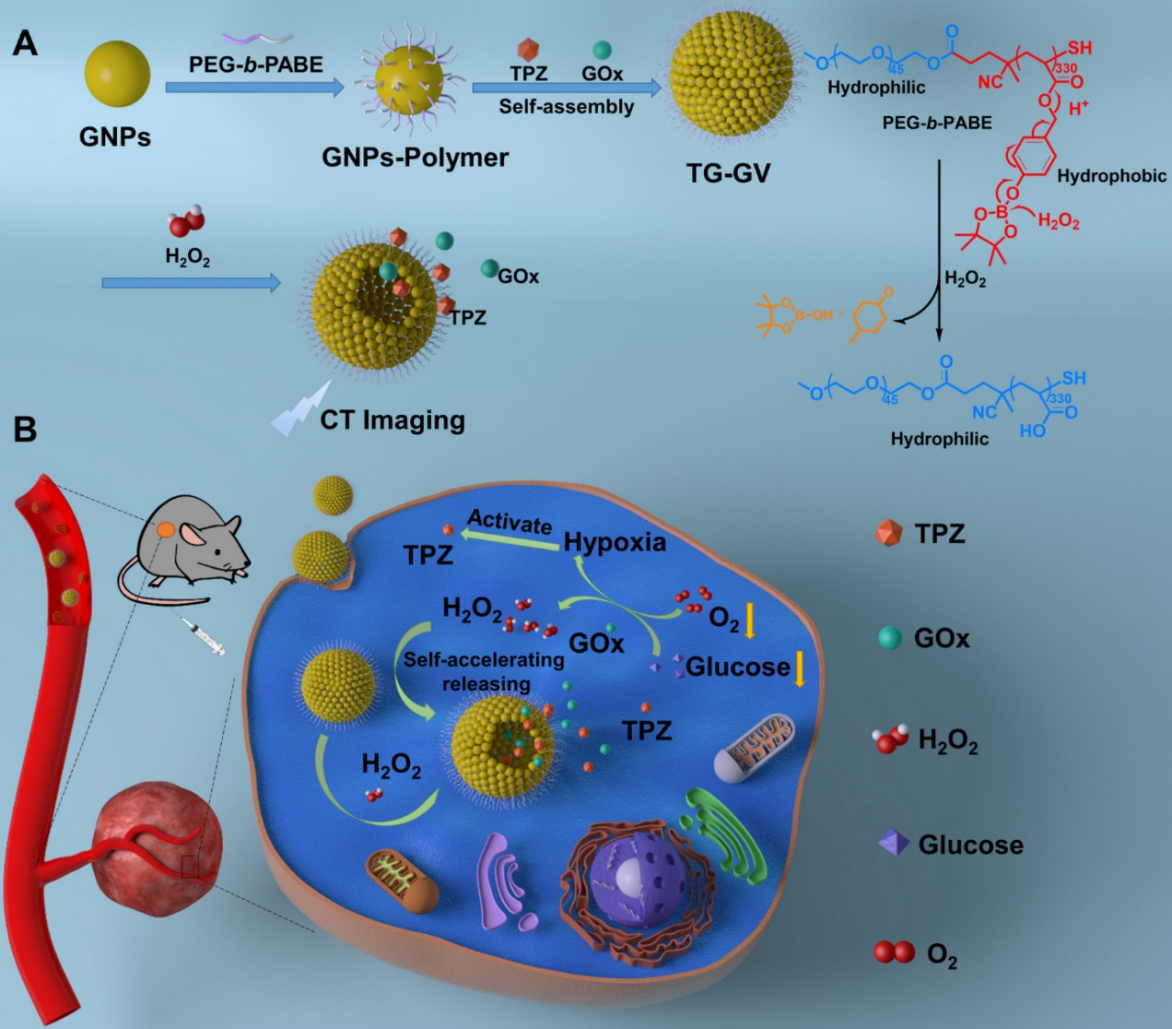
Schematic Illustration of (**A**) the fabrication of TG-GVs through the self-assembly of GNPs functionalized with amphiphilic block copolymers of PEG-*b*-PABE that are responsive to H_2_O_2_ and (**B**) the use of the TG-GVs for synergistic chemo/starving therapy via self-accelerated release of therapeutic compounds.

Herein, we report the design and fabrication of self-accelerated H_2_O_2_-responsive plasmonic GVs encapsulated with enzymes and therapeutic agents for computed tomography (CT) imaging and synergistic chemo/starving therapy of cancers. The monodisperse GVs are prepared by the self-assembly of GNPs grafted with amphiphilic block copolymers of polyethylene glycol-*b*-poly (4-(4,4,5,5-Tetramethyl-1,3,2-dioxaborolan-2-yl)benzyl acrylate) (PEG-*b*-PABE) that are responsive to H_2_O_2_ (Scheme 1) 1. Both hypoxia pro-drug tirapazamine (TPZ) and glucose oxidase (GOx) are simultaneously encapsulated in the GVs during the assembly process. In response to H_2_O_2_ in tumor, the TPZ- and GOx-loaded GVs (TG-GVs) dissociate to release payloads due to the oxidation of the hydrophobic phenyl boric ester groups of PEG-*b*-PABE into hydrophilic acrylic acid; otherwise, the encapsulated molecules retain in the vesicles without notable leakage for days 56. The released GOx catalyzes the oxidation of glucose by oxygen in the tumor tissue to enhance the degree of hypoxia. As a result, the released TPZ molecules are activated to produce highly toxic free radicals in tumors for chemotherapy 57. In addition, H_2_O_2_ generated in the redox reaction, in turn, accelerates the dissociation of vesicles to speed up the release rate of cargoes. The TG-GVs serve as contrast agent for CT imaging of tumors *in vivo* 58-60, which provides valuable information on the accumulation and metabolism process of the nanomedicine in tumor area. Furthermore, the TG-GVs exhibit superior tumor inhibition efficacy in murine 4T1 breast tumor-bearing mice, owing to the synergistic effect of chemo/starvation therapy. The nanoplatform may find applications in managing tumors deeply trapped in viscera or other important tissues that are not compatible with external stimulus (e.g. light).

## Methods

### Materials

Chloroauric acid (HAuCl_4_·4H_2_O), Sodium Citrate (C_6_H_5_O_7_Na_3_.2H_2_O), 4-(Hydroxymethyl) phenylboronic acid pinacol ester, acryloyl chloride, n-butylamine, hexadecyltrimethylammonium bromide (CTAB), azobis(isobutyronitrile), triethylamine (TEA), tirapazamine (TPZ), glucose oxidase (GOx), N,N-dimethylformamide (DMF), tetrahydrofuran (THF), dichloromethane(DCM), anisole, methanol, 3-(4,5-dimethyl-2-thiazolyl)-2,5-diphenyl-2-H-tetrazolium bromide-Thiazolyl Blue Tetrazolium Bromide (MTT) and all other chemical reagents were purchased from Sigma-Aldrich without any other purification process. Dulbecco's modified Eagle's medium (DMEM), Hoechst 33342, Amplex® Red, Image-iT™ Green Hypoxia Reagent and other biological reagents were purchased from Thermo Fisher Scientific.

### Synthesis of PEG_45_-*b*-PABE_330_

PEG_45_-*b*-PABE_330_ was synthesized by reversible addition-fragmentation chain transfer (RAFT) polymerization. The synthetic procedure is illustrated in Figure S1. The macromolecule chain transfer agent (PEG-CTA, 20.83 mg, 10.4 mmol, synthesized as reported previously 61), ABE (1.05 g, 3.64 mol, see supplementary information experimental section and Figure S2) and AIBN (0.34 mg, 2.07 mmol) were dissolved in anisole (3 mL), followed by deoxidizing for 30 min and placed in a pre-heated oil bath (85 °C) under stirring. After the reaction proceeded for 24 h, the solution was cooled under an ice bath to stop the reaction. Methanol was added to precipitate the polymer. The crude product was dissolved in THF and precipitated in methanol for 3 times to produce the pure PEG_45_-*b*-PABE_330_-CTA (505 mg). The as-synthesized PEG_45_-*b*-PABE_330_-CTA was treated by n-butylamine (25 μL) in THF (2 mL) to convert the dithiocarbonate into thiol group under stirring for 30 min. The thiol-terminated PEG_45_-*b*-PABE_330_ was obtained by precipitating the polymer in methanol for three times and dried under vacuum for 3 days. The ^1^H NMR spectral of the PEG_45_-*b*-PABE_330_ shows the resonance of the aromatic hydrogen (-CH-, 7.75-7.65 ppm and 7.23-7.10 ppm) and the methylene hydrogen (-CH_2_-, 5.00-4.70 ppm) in the phenylboronic acid pinacol ester, the resonance of the methylene hydrogen (-CH_2_-, 3.70-3.60 ppm) in the PEG chain, the resonance of the hydrogen in the acrylic acid backbone (-CH_2_-CH-, 2.40-1.50 ppm) and the hydrogen of the methyl in the pinacol ester (-CH_3_, 1.33-1.18 ppm) (Figure S3).

### Surface functionalization and self-assembly of GNPs

To graft polymers onto the surface of GNPs, a solution of PEG_45_-*b*-PABE_330_ in DCM (10 mg/mL, 1.6 mL) was injected into a solution of freshly prepared GNPs to produce emulsions. The emulsions were stirred vigorously overnight at room temperature. Ethanol was added to break down the emulsions, followed by separating the water phase from the lower organic layer in a funnel. The GNPs in the organic layer were precipitated by ethanol and centrifugation at 6000 rpm for 10 min to remove un-tethered polymers and re-dissolved in THF. The GNPs were further purified by centrifugation at 14000 rpm for 6 times to remove free polymers. To assemble polymer-grafted GNPs into TG-GVs, a 500 μL solution of GNPs in THF was dropwise added into a 1 mL solution of TPZ (1.5 mg mL^-1^) and GOx (1.5 mg mL^-1^ GOx) in buffer (HEPES 5 mM, pH 7.4) under gentle stirring in a glass vial. The solution was stirred overnight to evaporate the solvent of THF. The TG-GVs were centrifuged for 3 times to remove unloaded GOx and TPZ.

### Characterization

The chemical structure of PEG_45_-*b*-PABE_330_ was characterized by ^1^H NMR (Figure S3). The TG-GVs were imaged by Hitachi SU-70 Schottky field emission gun scanning electron microscope (FEG-SEM) and Hitachi JEOL JEM-2100 high resolution transmission electron microscopy (HR-TEM). The element mapping of vesicles was obtained by HR-TEM. The UV-Visible absorption of vesicles was characterized by Varian Cary 100 UV-vis Spectrophotometer. Zetasizer Nano ZS90 was used to measure the hydrodynamic diameter and zeta potential of all the samples.

### Controlled release of payload from TG-GVs

The H_2_O_2_-triggered release of payload from TG-GVs was investigated by treating TG-GVs (1 mg mL^-1^) with different concentrations of H_2_O_2_ (100 μM, 500 μM and 1 mM; HEPES 10 mM, pH 7.4) under gentle stirring at 37 °C. The accelerated release of payload from TG-GVs in the presence of glucose was tested by treating TG-GVs (1 mg mL^-1^) with different solutions: H_2_O_2_ 100 μM for TG-GV+H_2_O_2_ group; H_2_O_2_ 100 μM, glucose 1 mg mL^-1^ for TG-GV+H_2_O_2_+Glucose group; glucose 1 mg mL^-1^ for TG-GV+Glucose group. To quantify the mount of H_2_O_2_ generated through the GOx-catalyzed reaction, aliquots from TG-GV+H_2_O_2_+Glucose groups at different time point were taken and diluted for 100 times for the Amplex® Red assay. For comparison, free GOx (38.2 μg mL^-1^) was dissolved in an aqueous buffer solution of glucose (1 mg mL^-1^) containing 100 μM H_2_O_2_. The produced H_2_O_2_ was quantified by the Amplex® Red assay.

### Cytotoxicity of TG-GVs and control groups

Cytotoxicity of TG-GVs and control groups (GVs, T-GVs and G-GVs) was evaluated by the MTT assay. The 4T1 or L929 cells were planted in 96-well plates at a density of 1×10^4^ per well in 100 μL of DMEM (Dulbecco's modified Eagle's medium) with high glucose which contains 10% FBS for 24 h. The 4T1 cells were incubated in a 200 μL solution of culture medium containing different concentrations of GVs, T-GVs, G-GVs and TG-GVs (0, 4, 8, 12, 16 and 20 μg mL^-1^) for 24 h. For the Phorbol 12-myristate 13-acetate (PMA) group, the 4T1 cells were pre-treated by culture medium containing 200 nM of PMA for 1 h and then incubated in a new culture medium containing different concentrations of GVs, T-GVs, G-GVs and TG-GVs (0, 4, 8, 12, 16 and 20 μg mL^-1^) for 24 h. For the catalase group, the 4T1 cells were pre-treated by culture medium containing catalase (500 units mL^-1^) for 1 h and then incubated in a culture medium containing different concentrations of GVs, T-GVs, G-GVs and TG-GVs (0, 4, 8, 12, 16 and 20 μg mL^-1^) for 24 h. For the group without glucose, the 4T1 cells were incubated in a culture medium containing different concentrations of GVs, T-GVs, G-GVs and TG-GVs (0, 4, 8, 12, 16 and 20 μg mL^-1^) but without glucose for 24 h. For the L929 group, L929 cells were incubated in culture medium containing different concentrations of GVs, T-GVs, G-GVs and TG-GVs (0, 4, 8, 12, 16 and 20 μg mL^-1^) for 24 h. Then, a 20 μL of MTT solution (5 mg mL^-1^) was added into every well and the plate was incubated for 4 h, followed by replacing the medium with 100 μL DMSO to completely dissolve the precipitant. The absorbance of the solution at 492 nm and 630 nm was measured by a microplate reader and used for calculating the cell viability. For live/dead assay, cells were seeded in 24-well plate at 8 × 10^4^ per dish and incubated overnight. The medium was replaced by fresh medium (500 μL) containing GVs, T-GVs, G-GVs or TG-GVs (20 μg mL^-1^). The cells were incubated for 24 h, followed by washing with PBS for 3 times. The cells were then stained with calcein AM and propidium iodide before imaging by fluorescence inverted microscope.

### *In vitro* cell imaging

To image GOx-induced hypoxia in cells, the cells were incubated in confocal dishes at 2 × 10^4^ per dish overnight and then in a fresh medium (1 mL) containing 5 μM of Image-iT™ Green Hypoxia Reagent for 30 min. After replacing the medium with freshly prepared medium containing TG-GVs or GVs (1 mL, 10 μg mL^-1^), the dishes were sealed and placed in the incubator for 5 h. The cells were then stained by Hoechst 33342 and washed by PBS for 3 times before imaging by confocal laser scanning microscope (CLSM). To image H_2_O_2_ generated in cells, the cells were incubated in confocal-dishes at 2 × 10^4^ per dish overnight. After replacing the old medium with fresh one (1 mL) containing 5 μM of Horseradish peroxidase (HRP), the cells were incubated for another 30 min. The cells were then treated with new medium (1 mL) with 50 μM of Amplex® Red and 10 μg mL^-1^ TG-GVs for 5 h. The cells were imaged by CLSM after being stained by Hoechst 33342 and washed by PBS for 3 times.

### *In vivo* CT imaging

A Micro-computed tomography (NEMO®Micro CT, NMC-100, PINGSENG Healthcare, China) with tube voltage 90 kV, tube current 0.08 mA and slice thickness 0.16 mm was used for CT imaging of TG-GVs *in vivo*. Female balb/c nude mice (Shanghai SLAC Laboratory Animal Co., Ltd.) were used in all the animal studies. The animal experiments were performed using standard protocols approved by the Scientific Investigation Board of East China University of Science and Technology. The Balb/c nude mice with tumor xenograft (4T1 mammary orthotopic tumor model) were injected with a dose of 300 mg kg^-1^. The mice were scanned to construct images at the time points (6 h, 12 h, 24 h, 36 h and 48 h) of Pre-injection. The gray scale in images was converted into the CT values. Solutions of TG-GVs with different concentrations (2, 4, 6, 8 and 10 mg mL^-1^) were placed in 200 μL Eppendorf tubes and scanned by Micro CT imaging system for the production of a standard calibration curve.

### *In vivo* Tumor Therapy

Female balb/c nude mice (6 weeks old) with 4T1 tumor xenograft (orthotopic tumor model) were randomly divided into six groups (n=5) when the sizes of tumors reached 100 mm^3^. Each group of mice received systematic administration of phosphate buffered saline (1 X, 200 μL), TPZ (3 mg kg^-1^, 200 μL), GVs (100 mg kg^-1^, 200 μL ), T-GVs (100 mg kg^-1^, 200 μL), G-GVs (100 mg kg^-1^, 200 μL) and TG-GVs (100 mg kg^-1^, 200 μL) for a treatment period of 15 days, respectively. The mice were injected every 3 days and the tumor sizes and the body weight were recorded every 3 days. The photos of mice were taken on day 1, day 8 and day 15. After the therapy, mice were dissected and the tumors were weighed and arranged on a plate for taking photos. All the dissected organs were frozen, sliced, and stained by hematoxylin and eosin for tissue analyzing. Tumor slices were stained by terminal deoxynucleotidyl transferase (TdT) and 2'-Deoxyuridine 5'-Triphosphate (dUTP) for TUNEL assay, and treated by HIF-1α immunofluorescence assay for hypoxia condition detection.

## Results and Discussion

### Synthesis and characterization of TG-GVs

**Figure 1:**
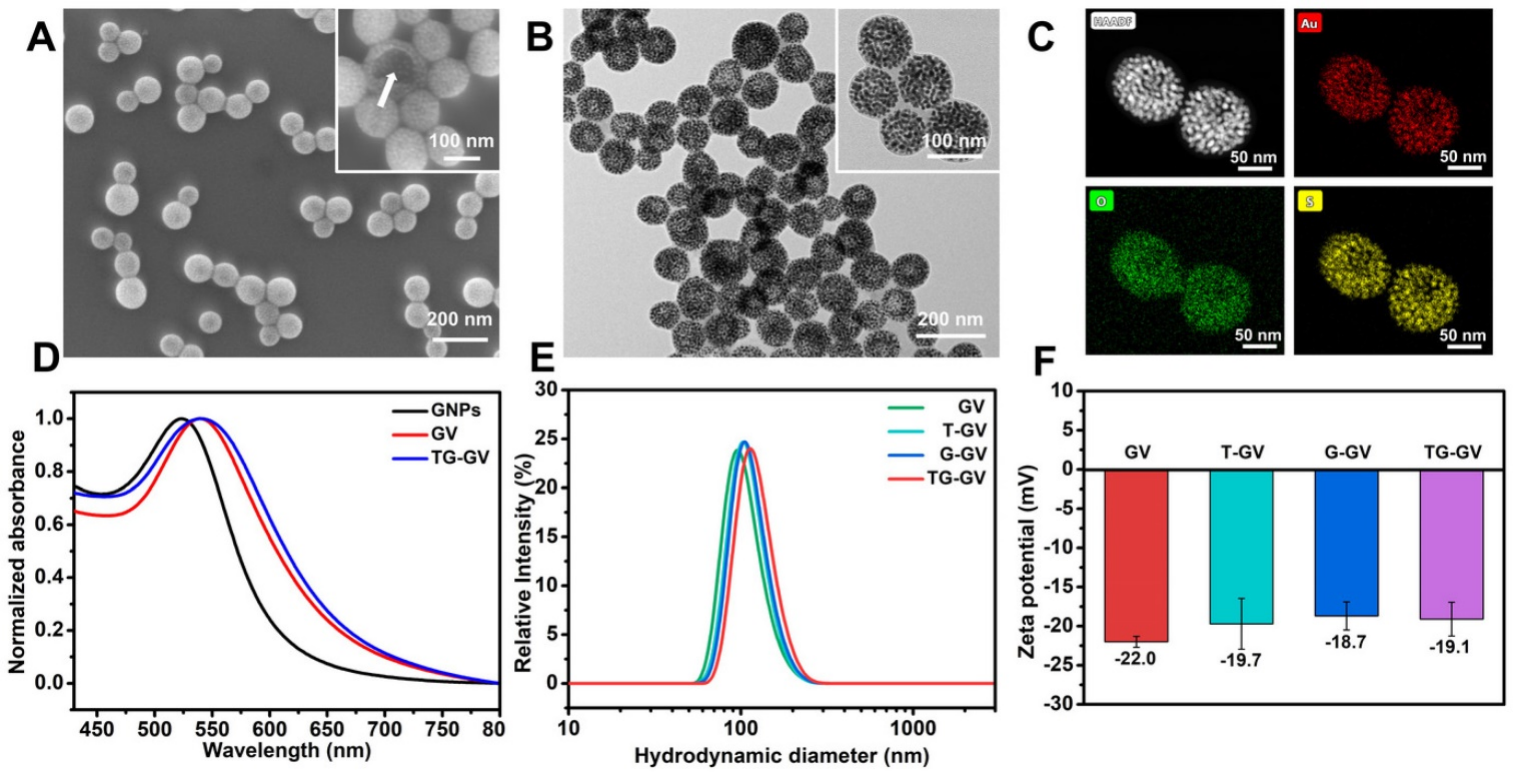
Characterization of TG-GVs assembled from PEG_45_-*b*-PABE_330_-grafted GNPs. (**A,B**) SEM (A) and TEM (B) images of TG-GVs. Insets are magnified images of the vesicles. The arrow in the inset of (A) pointed to the broken membrane of a vesicle. (**C**) STEM-HAADF image of GVs and the corresponding EDS element mapping. (**D**) UV-Vis spectra of GNPs, GVs and TG-GVs dispersed in water. (**E,F**) DLS analysis (E) and zeta potential measurement (F) of GVs, T-GVs, G-GVs and TG-GVs.

Scheme 1 illustrates the preparation of TG-GVs loaded with TPZ and GOx and their use in chemo/starving therapy. The amphiphilic block polymers of thiol-terminated PEG_45_-*b*-PABE_330_ were synthesized by reversible addition-fragmentation chain transfer (RAFT) polymerization (Figure S1-S3). In the presence of H_2_O_2_, the hydrophobic ABE block becomes hydrophilic in an aqueous environment, due to the conversion of the phenyl borate groups into acrylic acid (Figure S4). The structure and molecular weight of the polymer were determined by ^1^H NMR (Figure S2-S3). Monodispersed 7 nm-sized GNPs capped with cetyltrimethyl ammonium bromide (CTAB) were synthesized by a seeding growth method 62 and grafted with PEG_45_-*b*-PABE_330_ copolymers through ligand exchange (Figure S5-S6). The copolymer-grafted GNPs were dispersed in tetrahydrofuran (THF) which is a good solvent for the copolymers. Their self-assembly into nanosized vesicles was triggered by adding water into the dispersion, followed by slow evaporation of THF. To load TPZ and GOx in the hollow cavity - an aqueous environment - of the vesicles, TPZ and GOx were dissolved in water for the self-assembly. Scanning electron microscope (SEM) and transmission electron microscope (TEM) images show that the nanovesicles were composed of a single layer of GNPs in the vesicular membrane and a hollow cavity (Figure 1A-B and S7). The dense packing of impermeable GNPs in the membrane reduces the diffusion of loaded therapeutic compounds across the vesicular membrane, thus enhancing the retention ability of payloads. The vesicles had a uniform diameter of 100.3 ± 8.1 nm by analyzing SEM/TEM images. The existence of Au, O and S in the element mapping of the nanostructure indicates the presence of GNPs and PEG-*b*-PABE copolymers in the vesicles (Figure 1C). The TG-GVs exhibited a strong absorption at the wavelength of ~580 nm where light cannot penetrate into soft tissues deeply, which makes them not ideal for *in vivo* photo-based imaging or therapy (Figure 1D). The hydrodynamic diameter of TG-GVs was measured to be 129.5 ± 35.0 nm by dynamic light scattering (DLS) (Figure 1E). The slightly larger value obtained from DLS than TEM/SEM suggests that the outer surface of the vesicles is covered with a layer of hydrophilic polymer brushes for stabilization. The zeta potential of TG-GVs was measured to be -22.0 mV (Figure 1F). We presume that the negative charges are originated from the hydrolysis of small amount of borate ester groups 63, which would not affect the responsiveness of vesicles to H_2_O_2_. The encapsulation of TPZ or GOx in the vesicles did not drastically alter the size and surface properties of vesicles (Figure 1E-F).

### H_2_O_2_-triggered release of payload from TG-GVs

**Figure 2:**
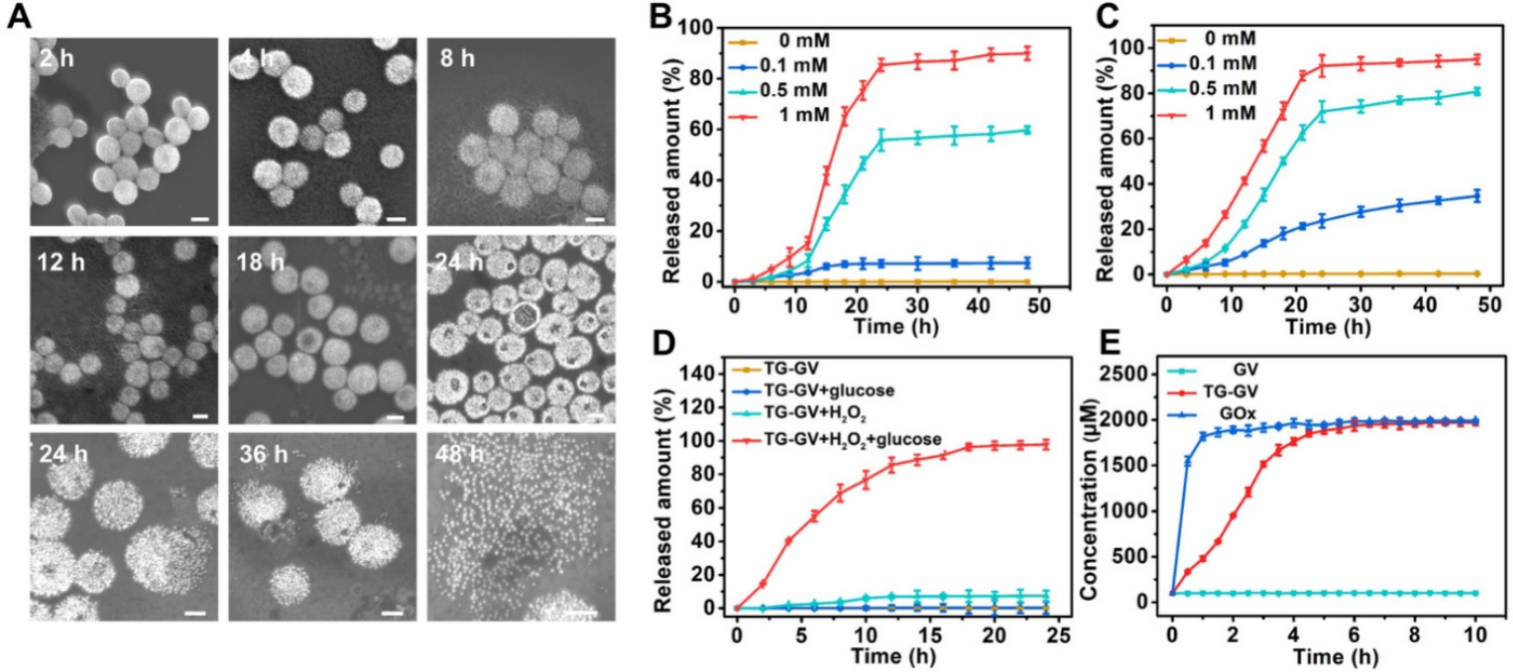
The controlled release of payloads from TG-GVs in response to H_2_O_2_. (**A**) SEM images showing the degradation process of TG-GVs under 1 mM H_2_O_2_ at the time points of 2 h, 4 h, 8 h, 12 h, 18 h, 24 h, 36 h and 48 h. The scale bar is 100 nm. (**B,C**) The release of TPZ (B) and GOx (C) from TG-GVs at different *c*_H2O2_. (**D**) The release of TPZ from TG-GVs with or without glucose in the system (H_2_O_2_: 0.1 mM, glucose: 1 mg mL^-1^). (E) The variation in the concentration of H_2_O_2_ over time during the oxidation of glucose by free GOx (GOx: 38.2 µg mL^-1^, glucose: 1 mg mL^-1^) or GOx released from TG-GVs (H_2_O_2_: 0.1 mM, glucose: 1 mg mL^-1^).

The TG-GVs are stable in water, cell medium and plasma of rats without any aggregation or morphological change for at least three weeks (Figure S8), thanks to the combination of steric and electrostatic stabilization. In the presence of H_2_O_2_, the hydrophobic PABE block turned into hydrophilic, triggering the dissociation of the vesicles. Figure 2A shows the degradation process of TG-GVs in water solution containing 1 mM of H_2_O_2_. At 2 h, vesicles remained largely intact. At 4 h, the membrane of the vesicles started to gradually swell and the interparticle distance between GNPs increased, as indicated by an increase in the average diameter of vesicles from 100 nm to 140 nm. The swelling of vesicles became more and more obvious at the time point of 8 h and 12 h. With time, visible holes appeared on the vesicular membranes and eventually the vesicles completely dissociated into individual GNPs at 36 h. The complete disassembly of the vesicles into small GNPs with diameter smaller than the kidney filtration threshold may promote the renal elimination of the vesicles from the body after their use *in vivo*.

We studied the retention ability of TPZ and GOx inside TG-GVs by dialyzing the vesicles against water. Figure S9 shows that both molecules stayed in TG-GVs without obvious leakage for at least three weeks. We further evaluated the controlled release of payloads from TG-GVs in response to H_2_O_2_. The loading amount of TPZ and GOx in the vesicles was measured to be 3.42% and 3.82%, when a 1.5 mg mL^-1^ concentration of both TPZ and GOx was used in the self-assembly process (Figure S10). The release rate increased with increasing the concentration (*c*_H2O2_) of H_2_O_2_ (Figure 2B-C). At *c*_H2O2_ ≤ 100 μM, the release of payloads was slow, with a ~5% of TPZ and ~31 % of GOx released at 24 h. At *c*_H2O2_ = 500 μM, ~55% of TPZ and ~65% of GOx were released at 24 h. The release rate (82% for TPZ and 85% for GOx at 24 h) was even higher at *c*_H2O2_ = 1 mM. In all the cases, the release of GOx was faster than TPZ, probably due to the hydrophobic interaction between TPZ and hydrophobic PABE block of the copolymers in the membrane.

The results indicate that the release kinetics was not satisfactory under a common level of H_2_O_2_ concentration (~100 μM) in tumor. The encapsulation of GOx in the TG-GVs enabled a self-accelerating mechanism for triggering the release of therapeutic compounds. The GOx released from vesicles effectively catalyzed the oxidation of glucose in tumor to produce a large amount of H_2_O_2_ which accelerated the dissociation of vesicles to release more TPZ and GOx. Figure 2D shows that 84% of loaded TPZ was released from TG-GVs at 15 h at *c*_H2O2_ = 100 μM in the presence of 1 mg mL^-1^ glucose. In contrast, no cargoes leaked out from the vesicles when there is only glucose but without H_2_O_2_ to initiate the release process. We quantified the production of H_2_O_2_ from enzymatic oxidation by Amplex® Red assay, when the total amount of both GOx and glucose was the same for the free GOx and TG-GV systems. The concentration of H_2_O_2_ gradually increased and reached a plateau of 1.8 mM for both systems, although the production rate of H_2_O_2_ in the TG-GV system was slower than that of the free GOx system due to the gradual release of loaded GOx from TG-GVs (Figure 2E and S11). This result confirms that the process of encapsulating and releasing GOx in TG-GVs did not obviously affect the enzymatic activity of GOx.

### *In vitro* cytotoxicity

**Figure 3:**
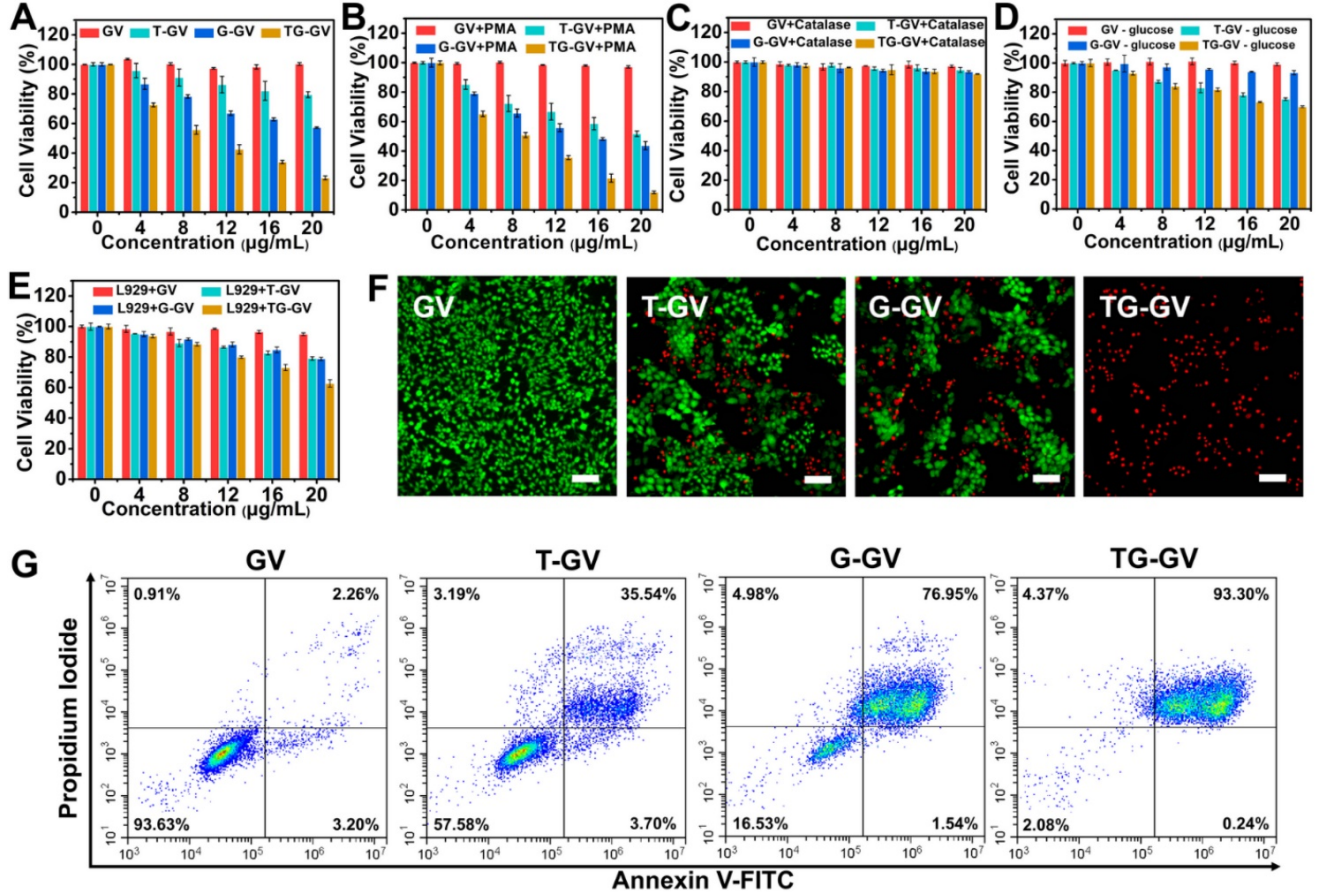
*In vitro* inhibition of cell growth by GVs, T-GVs, G-GVs and TG-GVs. (**A**) 4T1 cells treated by the vesicles. (**B**) 4T1 cells pretreated by PMA and then treated by the vesicles. (**C**) 4T1 cells pretreated by catalase to consume endogenous H_2_O_2_ and then treated by vesicles. (**D**) 4T1 cells treated by the vesicles in cell medium without glucose. (**E**) L929 cells treated by vesicles. (**F**) Live/dead assay of cells pre-treated with vesicles. Live cells were stained with calcein AM (green) while dead cells were stained with propidium iodide (red). The scale bar is 200 µm. (**G**) apoptosis/necrosis flow cytometry analyses of 4T1 cells after 24 h exposure to the vesicles.

We evaluated the *in vitro* toxicity of TG-GVs by methyl thiazolyl tetrazolium (MTT) assay using GVs, T-GVs and G-GVs as control groups (Figure 3A-E). Tumor cells produced H_2_O_2_ at a rate of 0.5 nmol/10^4^ cells/h, which is sufficient for initiating the self-accelerating release of drugs from TG-GVs 2. In the experiment, 4T1 cells were treated with different groups of samples for 24 h. Figure 3A shows that the GVs were non-toxic to 4T1 cells, indicating the excellent bio-compatibility of the GNPs and copolymers. T-GVs and G-GVs showed comparable cytotoxicity at a concentration above 4 μg mL^-1^. In contrast, TG-GVs exhibited a significantly higher cytotoxicity to 4T1 cells than all the control groups, due to the combination effect of TPZ and GOx. The continuous consumption of glucose and oxygen in the cancer cells through the GOx-catalyzed reaction starved the tumor cells and created a hypoxia condition in the tumors. The latter further activated the hypoxia effect of pro-drug TPZ to generate toxic oxidizing radical species that break down DNA double strands, leading to chromosome aberrations. Furthermore, the continuous production of H_2_O_2_ by GOx, in turn, accelerated the release of loaded cargos and the inhibition of tumor cell proliferation. Phorbol 12-myristate 13-acetate (PMA) was used to induce the cancer cells to generate more H_2_O_2_ to further increase the inhibition effect of cell proliferation 64-66. A concentration of PMA below 600 nM did not show obvious cytotoxicity to 4T1 cells (Figure S12.). We, thus, used 200 nM of PMA to pre-treat the 4T1 cells 64. Figure 3B shows that the inhibition rates of cell proliferation drastically increased for all the groups of T-GVs, G-GVs and TG-GVs, compared with the non-treated groups. The result confirms that the elevated initial H_2_O_2_ level effectively accelerated the release rate of TPZ and GOx and reduced the viability of 4T1 cells. To quantitatively analyze the combinational effects of TPZ and GOx on the inhibition of cell growth, the combination index (CI) were determined by the Chou-Talalay method 67. The IC_50_ of T-GVs, G-GVs and TG-GVs in the MTT assay of PMA pre-treated 4T1 cells were 22.1 μg mL^-1^, 15.3 μg mL^-1^ and 7.9 μg mL^-1^ respectively. The CI value was determined to be 0.872, indicating the realization of a synergistic effect of the chemo/starvation therapy (Supplementary Information).

We used catalase to consume the endogenous H_2_O_2_ in 4T1 cells before the cells were treated with different vesicles. Figure 3C indicates that the inhibition rates of cell proliferation by T-GVs, G-GVs and TG-GVs significantly decreased after the addition of catalase (Figure 3A). The result indicates that the consumption of endogenous H_2_O_2_ prevents the release of cargos from the vesicles. We studied the effect of glucose absence on the cytotoxicity of our vesicles in the MTT assay, as the self-accelerating release of payload relies on the glucose oxidation reaction. As shown in Figure 3D, the inhibition rates of cell proliferation by G-GVs and TG-GVs significantly decreased, compared with the non-treated groups (Figure 3A), while the cell viability of T-GVs group remained the same. This result indicates the strong dependence of the cytotoxicity of G-GVs and TG-GVs on the presence of glucose in the system. The L929 cells (mouse fibroblast, normal cells) were then used as a model to test the toxicity of GVs, T-GVs, G-GVs and TG-GVs to normal cells. Figure 3E shows that the inhibition rates of cell proliferation of L929 cells are lower than 4T1 cells (Figure 3A). This could attribute to the less H_2_O_2_ produced by normal cells.

We investigated the efficiency of cell inhibition by TG-GVs and control groups using live/dead cellular assays (Figure 3F). The live/dead cells were stained by calcein-AM and propidium iodide (PI), respectively. The empty GVs without any drugs (control group) did not show detectable red fluorescence, suggesting the good bio-compatibility of GVs. In contrast, the cells treated with TG-GVs showed a ~100% of apoptosis of cells, which is significantly higher than 39% for T-GV and 48% for the G-GV group. We further studied the cell-killing ability of our vesicles by adding exogenous H_2_O_2_ (100 μM) into the system before cellular uptake of the vesicles. Figure S13 shows that the percentage of apoptotic cells increased to 43% and 75 % respectively for the T-GV and G-GV groups, while the cell apoptosis remained 100% For the TG-GV group. This result further confirms that the increased release rate of TPZ and GOx from the vesicles induced by elevated H_2_O_2_ level reduces the viability of 4T1 cells. The cells were stained by annexin V-FITC and propidium iodide (PI) and the cell apoptosis induced by different vesicles was quantitatively analyzed using flow cytometry. Figure 3G shows that 35.54%, 76.95% and 93.30% of the 4T1 cells entered the late apoptosis stage after treated by T-GVs, G-GVs and TG-GVs, respectively. These data collectively demonstrated that TG-GVs exhibit high lethality to 4T1 cells due to the combination effect of self-accelerating release and synergistic chemo/starvation therapy.

### *In vitro* endocytosis, deoxygenation effect, H_2_O_2_ production and starvation effect

**Figure 4:**
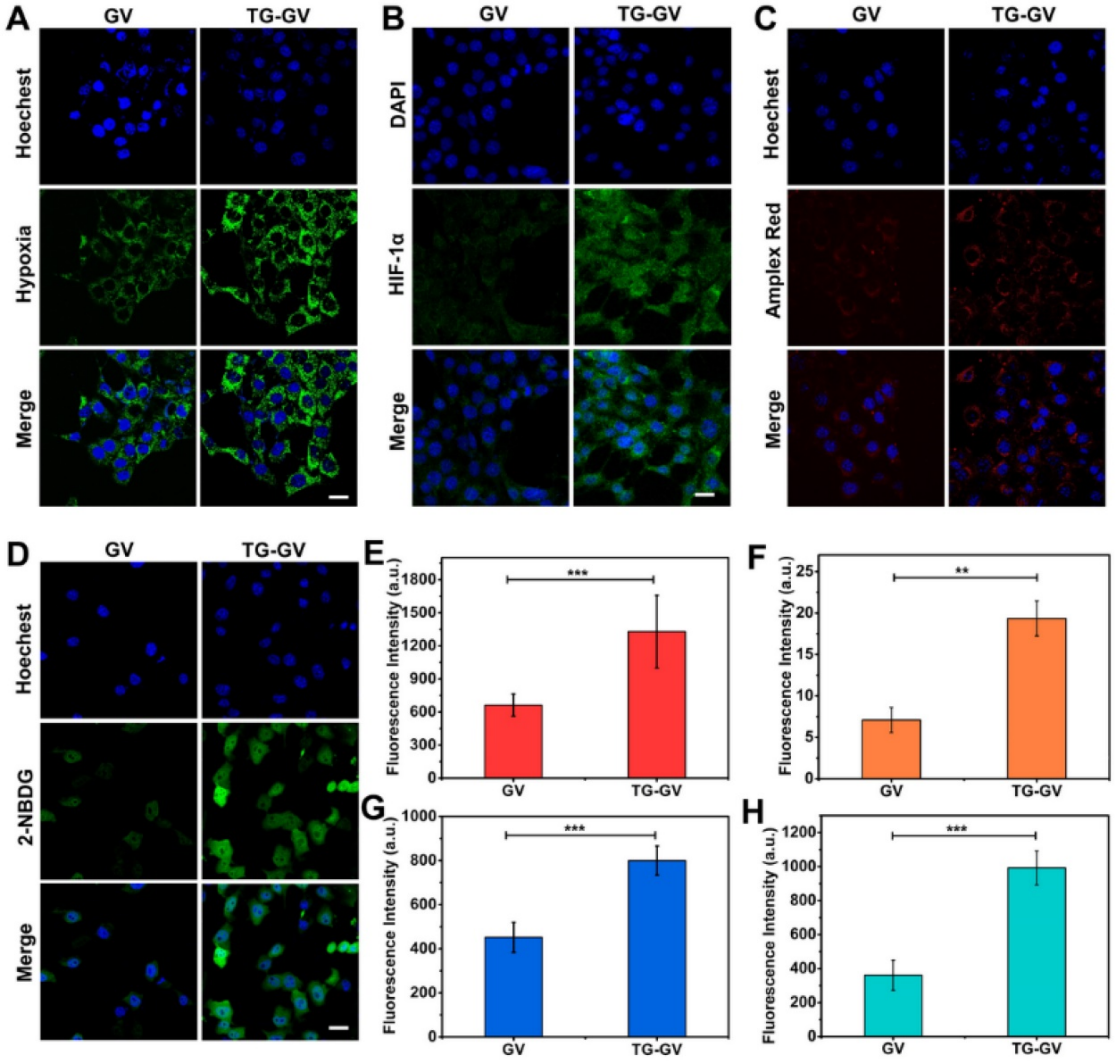
CLSM observation of cellular hypoxia conditions (**A**), HIF-1α expression levels (**B**), H_2_O_2_ levels (**C**) and glucose uptake conditions (starvation degree) (**D**) of 4T1 cells treated with GVs or TG-GVs. The scale bars are 25 µm. Mean fluorescence intensity of the cells treated with GVs or TG-GVs in hypoxia assay (**E**), HIF-1α immunofluorescent staining (**F**), H_2_O_2_ assay (**G**) and glucose uptake assay (**H**). Data are the means ± s.d. (n = 3, ***P < 0.001, **P < 0.01).

We used confocal laser scanning microscope (CLSM) to elucidate the endocytosis of TG-GVs by the cells, the effect of deoxygenation, high levels of H_2_O_2_ produced in the cells and the starvation effect induced by TG-GVs (Figure S14 and 4). First, the TG-GVs were marked by Rhodamine B (RhB) and then treated with 4T1 cells. Figure S14 shows the time-dependent endocytosis process of TG-GVs by 4T1 cells. Second, the 4T1 cells were treated with commercial hypoxia probe Image-iT™ Green Hypoxia Reagent, and then incubated with TG-GVs or GVs for 5 h in sealed dishes. The cells treated by TG-GVs exhibited stronger fluorescence intensity than those treated by GVs, indicating the enhanced hypoxia condition due to the oxygen consumption in the GOx-catalyzed reaction (Figure 4A and 4E). The hypoxia condition of the cells induced by TG-GVs were also confirmed by the HIF-1α immunofluorescence staining. As shown in Figure 4B and 4F, the expression of HIF-1α in cells treated by TG-GVs for 5 h were significantly higher than the GVs group, as indicated by a stronger green fluorescence for cells of this group. Third, the generation of H_2_O_2_ catalyzed by the released GOx was verified by using Amplex® Red as a probe. The 4T1 cells were pre-treated with horseradish peroxidase (HRP), washed by fresh medium and treated with Amplex® Red and TG-GVs or GVs for 5 h. The Amplex® Red can be oxidized by H_2_O_2_ to produce a fluorescent molecule of resorufin. Figure 4C and 4G show that the cells incubated with TG-GVs showed obvious red fluorescence, while the fluorescence in the GV group was negligible. The same effect of deoxygenation and high levels of H_2_O_2_ produced in the cells were also observed in cells treated by free GOx (Figure S15), confirming their origins from GOx encapsulated in the TG-GVs. Finally, the starvation effect induced by TG-GVs was examined by treating 4T1 cells with TG-GVs and fluorescent glucose analogue of 2-(N-(7-nitrobenz-2-oxa-1,3-diazol-4-yl)-amino)-2-deoxyglucose (2-NBDG). Figure 4D and 4H show that the cell fluorescence of the TG-GV group was significantly higher than that of the GV group. The higher glucose demand by the cells treated by TG-GVs indicates the starvation effect of 4T1 cells induced by TG-GVs.

### The use of TG-GVs as contrast agent for *in vivo* CT imaging

**Figure 5:**
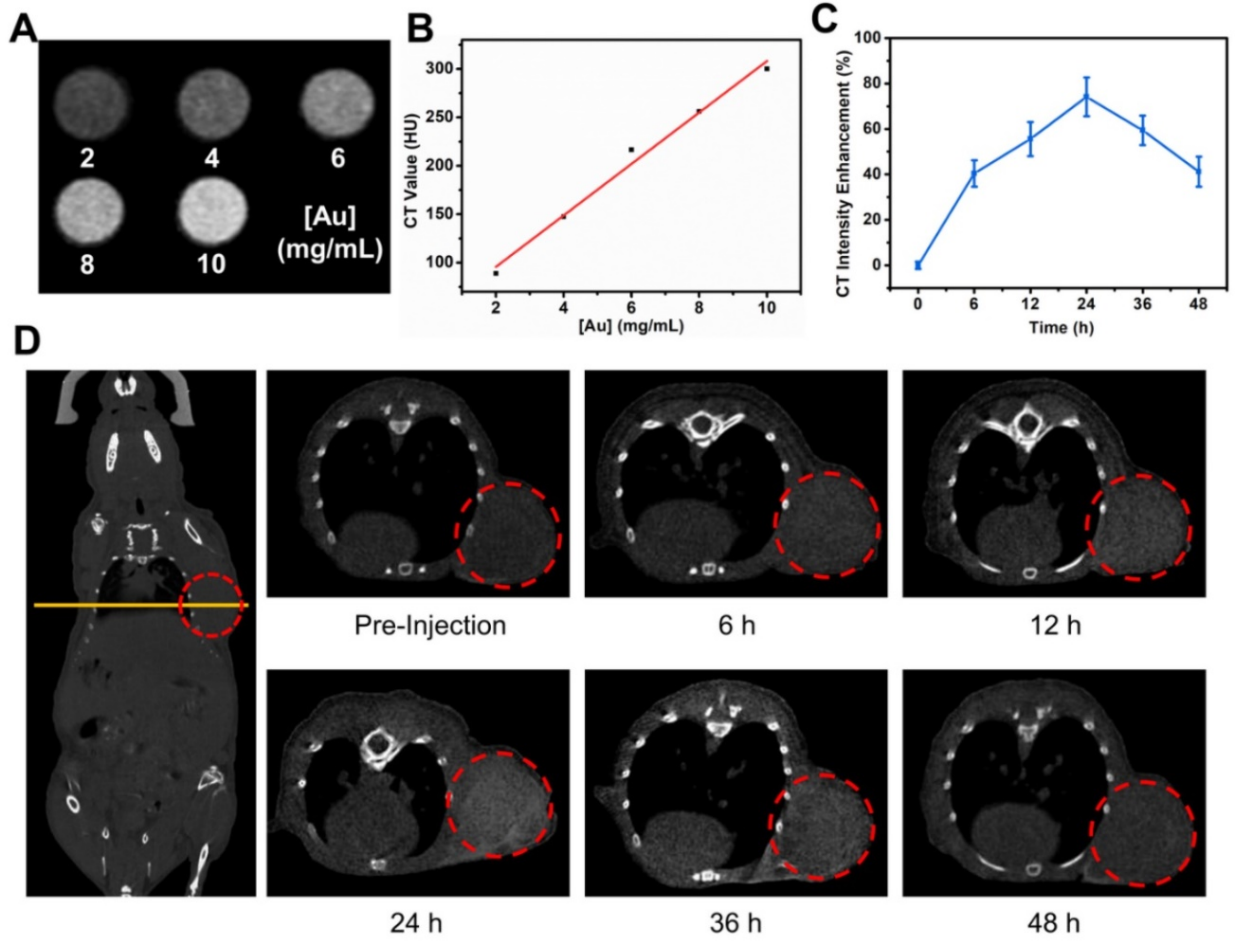
*In vivo* CT imaging of 4T1 tumor-bearing mouse injected with TG-GVs. (**A**) CT images and values (**B**) of TG-GVs with different concentrations (2, 4, 6, 8 and 10 mg mL^-1^). (**C**) CT intensity enhancement of the tumor sites over time (6 h, 12 h, 24 h, 36 h and 48 h). (**D**) Left: CT images of the whole mouse (coronal), right: CT images of tumor sties at pre-injection, 6 h, 12 h, 24 h, 36 h and 48 h. Tumor areas are marked by a red circle in each image.

Before the *in vivo* cancer therapy experiment, we investigated the use of TG-GVs as contrast agent for CT imaging. Figure 5A shows that the brightness of CT images increased with increasing the concentration of TG-GVs. The corresponding CT values (Hounsfield unit, Hu) were linearly correlated with the concentrations of Au (Figure 5B). The Balb/c mice with tumor xenograft (4T1 mammary tumor model) were administrated intravenously with TG-GVs. The CT values of the tumor sites increased and reached a peak value of 74.1% at 24 h after post-injection, followed by a gradual decrease in the next 24 h (Figure 5C-D). The accumulation of GVs at the tumor site was also confirmed by quantifying the concentrations of Au in tumors and other organs by inductively coupled plasma atomic emission spectrometry (ICP-AES) at 24 h post-injection (Figure S16). The results signify that the TG-GVs can serve as a CT contrast agent for *in vivo* tracking of TG-GVs, benefiting the optimization of treatment schedule.

### *In vivo* chemo/starvation therapy

**Figure 6:**
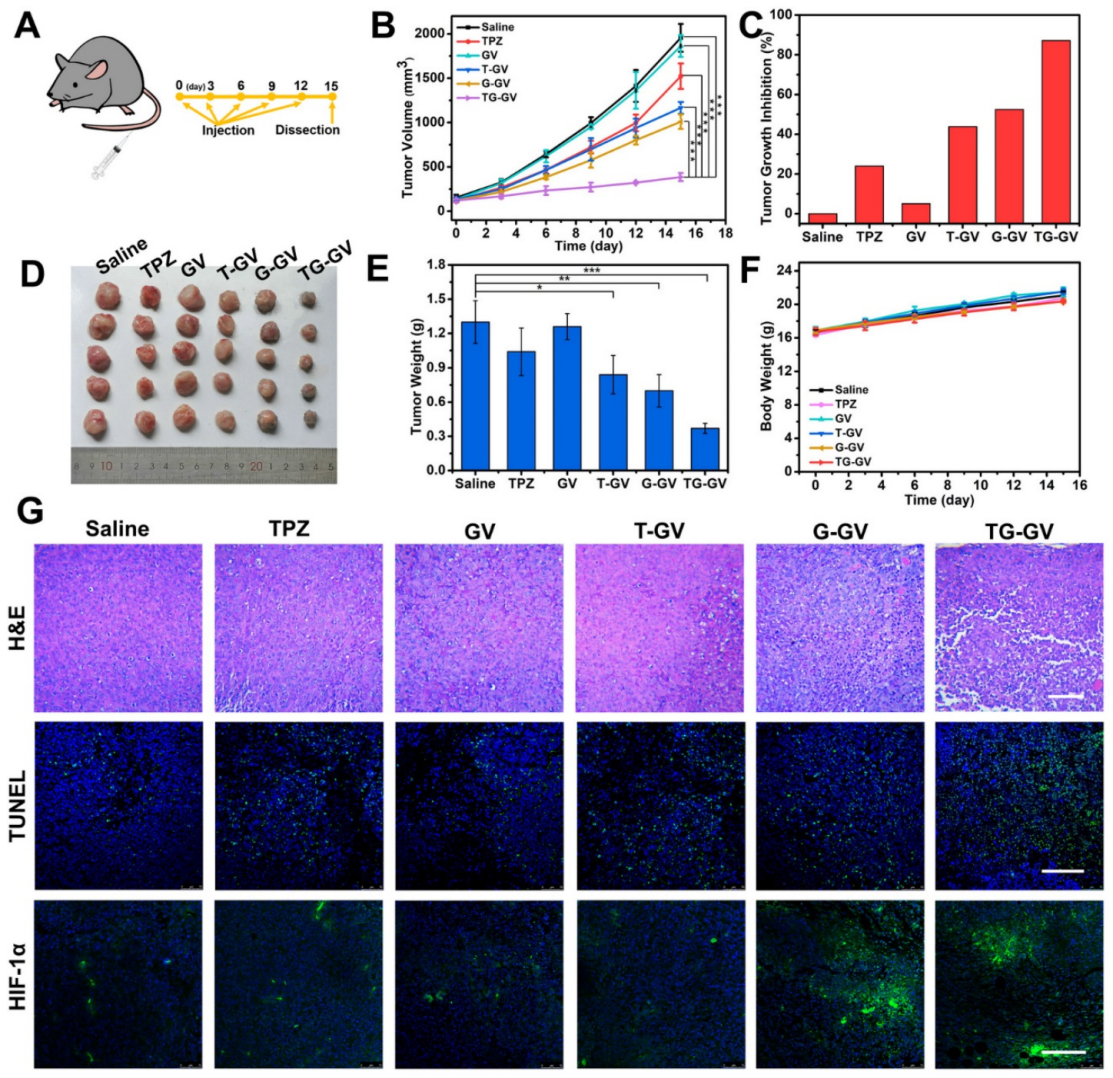
*In vivo* antitumor activities of TG-GVs and control groups (Saline, TPZ, GVs, T-GVs and G-GVs). (**A**) Injection schedule. (**B**) Tumor volume changes. Data are the means ± s.d. (n = 5, ***P < 0.001) (**C**) Tumor growth inhibition ratios (TGI) for different groups. (**D**) Photos and the (**E**) weights of the dissected tumors obtained after the 15-day therapy. (**F**) Body weight changes of mice during the treatment. (G) H&E staining and CLSM images of TUNEL assay and HIF-1α immunofluorescence staining of the dissected tumors after therapy. The scale bar is 200 µm in H&E staining and 150 µm in both TUNNEL assay and HIF-1α staining.

We further explored the *in vivo* therapeutic efficiency of TG-GVs for the treatment of tumors. Mice with tumor xenograft (4T1 tumor) received systematic administration of one of the treatment groups: Saline, TPZ, GVs, T-GVs, G-GVs and TG-GVs. During the 15-day period of therapy (Figure 6A), the group treated with TG-GVs showed a growth inhibition rate of up to 87.2%, owing to the synergistic effect of starvation and chemo-therapy (Figure 6B-C). The tumor growth inhibition rate (TGI) was found to be 24.0%, 43.8%, 52.5% for the groups treated with TPZ, T-GVs and G-GVs, respectively. In contrast, the Saline and GV groups showed a fast tumor growth and the tumor volume increased to 12 times of its origin size on day 15 (Figure 6B and S17). The antitumor efficacy was evaluated by Q value method (Supplementary Information) 68. The Q value for TG-GV group was 1.19, indicating a synergistic interaction of the chemo/starvation therapy. Figure S18 shows the representative photos of the mice in 1 day, 8 day and 15 day. After the treatment, the tumors and main organs including, heart, liver, spleen, lung, kidney were dissected and stained by hematoxylin and eosin (H&E) before photo-taking and weighting (Figure S19 and 6D-E). The tumor weight of TG-GV group was measured to be significantly lower than all the other groups, indicating the strongest antitumor effect of TG-GVs (Figure 6E). The body weight of all the mice steadily grew in the therapy process, implying the good biocompatibility of each material to the normal organs and tissues of the mice (Figure 6F). In the H&E staining of tumor slices, the TG-GV group exhibited more condensed nuclei, more vacuoles and shape-changed cells than all the other groups (Figure 6G). In terminal deoxynucleotidyl transferase uridine triphosphate dUTP nick-end labeling (TUNEL) assay, the most obvious apoptosis was observed in the TG-GV group, which is also correlated with the calculated apoptosis rate (Figure 6G and S20). Finally, in the HIF-1α immunofluorescence staining assay, tumor slices of the G-GV and TG-GV groups displayed the strongest fluorescence (i.e., the highest expression of HIF-1α in the tumor cells) (Figure 6G and S21). This result demonstrates the hypoxia effect induced by the released GOx from G-GVs or TG-GVs on the outcomes of treatment.

## Conclusions

In summary, we developed H_2_O_2_-responsive plasmonic GVs encapsulated with TPZ and GOx for CT imaging, and synergistic starvation and hypoxia-activated chemotherapy of cancer. In response to H_2_O_2_ presented in the tumor environment, TG-GVs released cargoes encapsulated in the vesicles in a self-accelerating manner to kill cancer cells with a high efficacy. The self-accelerating mechanism of releasing therapeutic compounds makes it possible for the use of TG-GVs in situation that requires a fast response of vesicles under a common level of H_2_O_2_ concentration (~100 μM) in tumor. In addition to serve as contrast agent for CT imaging, the TG-GVs effectively inhibited the growth of tumors *in vivo*. This nanoplatform may find application in effective tumor inhibition, especially for tumors deeply trapped in viscera or other tissues 69-72.

## Supplementary Material

Supplementary methods and figures.
