# Supplementary material for: Self-accelerating H2O2-responsive Plasmonic Nanovesicles for Synergistic Chemo/starving therapy of Tumors
Source: Theranostics. 2020 Jul 9;10(19):8691–704. doi: 10.7150/thno.45392 (PMC7392001; doi:10.7150/thno.45392)
Supplement: Supplementary file 1 — Supplementary methods and figures. [file thnov10p8691s1.pdf]

Supplementary Information

Self-Accelerating H_2O_2-responsive Plasmonic Nanovesicles for Synergistic Chemo/Starving therapy of Cancers

Yao Tang[1], Yuejia Ji[1], Chenglin Yi[2], Di Cheng[1], Bin Wang[1], Yun Fu[1], Yufang Xu[1], Xuhong Qian[1], Yahya E. Choonara[3], Viness Pillay[3], Weiping Zhu[1,]***, Yunen Liu[4,]**, Zhihong Nie[2,]*

1. State Key Laboratory of Bioreactor Engineering, Shanghai Key Laboratory of Chemical Biology, School of Pharmacy, East China University of Science and Technology, Shanghai 200237, China

2. State Key Laboratory of Molecular Engineering of Polymers, Department of Macromolecular Science, Fudan University, Shanghai 200438, China

3. Department of Pharmacy and Pharmacology, University of the Witwatersrand, Parktown 2193 Johannesburg, South Africa

4. Department of Emergency Medicine, the General Hospital of Northern Military Area, Laboratory of Rescue Center of Severe Trauma PLA, Shenyang 110016, China

Supplementary Experimental Section:

Synthesis of (4-(4,4,5,5-Tetramethyl-1,3,2-dioxaborolan-2-yl)benzyl acrylate) (ABE)

A solution of 4-(4,4,5,5-Tetramethyl-1,3,2-dioxaborolan-2-yl)benzenemethanol (2.34 g) in DCM (100 mL) was put in a 250 mL flask with an ice bath. Then 1.5 mL of triethylamine was added into the solution with stirring. 0.82 mL of acryloyl chloride in DCM (5 mL) was added into the solution dropwise with vigorous stirring. After adding acryloyl chloride, the reaction solution was stirring under ice bath for 10 min, followed by withdrawing the ice bath and stirring under room temperature for 20 min. Then, the solution was washed by saturated NaCl solution and dried by anhydrous Na_2SO_4, followed by evaporating the solvent to get crude product. The crude product was purified by column chromatography (PE/EA) to get pure colorless liquid ABE.

Synthesis of gold nanoparticles of 7 nm

GNPs of 7 nm were synthesized by a seed-induced growth method according to a published work with moderate modification. Briefly, 7.386 mg of $HAuCl_4$ (18.7 mmol) and 5.511 mg of trisodium citrate dehydrate (18.7 mmol) was dissolved in 75 mL of deionized water in a round-bottom flask. Then, 2.25 mL of iced $NaBH_4$ solution (100 mM) was injected into the solution with vigorous stirring to get the gold seeds. The seeds could be used in 2-5 h. The growth solution was prepared by adding 5.91 mg of $HAuCl_4$ (14.96 mmol) and 1.8 g of CTAB (4.94 mmol) into 60 mL of deionized water and heated to dissolve the CTAB. Then, 0.2 mL of ascorbic acid (0.1 M) was added into the growth solution with stirring, followed by injecting 20 mL of the seeds solution into the growth solution. After stirring for 1 h, 7 nm of CTAB-covered GNPs was obtained.

Stability of TG-GVs in water, cell medium and plasma

The stability of TG-GVs was tested in water, cell medium and plasma of rats. TG-GVs were dispersed in water, cell medium and plasma of rats. After 3 weeks, the vesicles were washed by pure water for 3 times. Then the vesicles were imaged by imaged by Hitachi SU-70 Schottky field emission gun Scanning Electron Microscope (FEG-SEM).

Loading content of TG-GVs

The standard curves of TPZ and GOx were shown in Figure S7. The concentration of TPZ was obtained by testing the absorbance of solution in 461 nm. For GOx, BCA assay was used to determine the concentration after the solution was pre-treated with MnO_2 to remove the H_2O_2 which

could severely affect the assay. To obtain the loading content, the solution of TG-GVs was centrifuged to remove the supernatant and added DMF to dissolve the precipitant. The solution was stirred for 30 min to totally break the vesicles and release the cargoes, followed by centrifuging to remove the GNPs. The supernatant was tested to obtain the loading content.

The retention ability of TPZ and GOx inside TG-GVs

The retention ability of TPZ and GOx inside TG-GVs was assessed by dialyzing the vesicles against water for 3 weeks. The outer solutions at 0 h, 4 h, 8 h, 12 h, 24 h, 2 days, 1 week, 2 weeks and 3 weeks were analyzed by UV-Visible Spectrophotometer to quantify the leaked TPZ at 461 nm. The solutions were analysed by BCA assay to determine the concentration of the leaked GOx.

Cell internalization

4T1 cells were incubated in confocal dishes at 2×10^4 per dish overnight. The cells were then incubated in fresh medium (1 mL) containing RhB marked TG-GVs (10 μg mL^{-1}) for 0.5 h, 1 h or 2 h. Before imaging by CLSM, the cells were washed by PBS for 3 times and stained by Hoechst 33342.

Exogenous H_2O_2 induced live/dead cellular assays

4T1 cells were incubated in 24-well plates at a density of 8×10^4 cells per well and then treated with H_2O_2 (100 μM) along with GVs, T-GVs, G-GVs and TG-GVs (20 μg mL^{-1}) for 24 h, followed by washing with PBS for 3 times. Then the cells were stained with calcein AM and PI for 30 min. The images of the live/dead cells were obtained by inverted fluorescence microscope.

Cell apoptosis assay by flow cytometry

4T1 cells were incubated in 6-well plates at a density of 2×10^5 per well and then treated with GVs, T-GVs, G-GVs and TG-GVs (20 μg mL^{-1}) for 24 h. Then the cells were digested by trypsin without EDTA, followed by centrifugation to wash the cells with iced PBS for 3 times. To stain the cells, Annexin V-FITC and PI buffer were used to treat cells for 15 mins. The cell apoptosis state was analyzed by flow cytometry (Beckman Coulter CytoFLEX).

HIF-1α immunofluorescence staining of 4T1 cells

4T1 cells were incubated in 6-well plates at a density of 2×10^4 per well overnight and then treated with GVs or TG-GVs (10 μg mL^{-1}) for 5 h. Then the cells were washed by PBS for 3 times, and then fixed with 1mL 4% formaldehyde solution, followed by permeabilizing by 0.5% Triton X-100 for 20 min, and blockage with 5% bovine serum albumin (BSA, 5%) for 30 minutes. After the

BSA solution was removed, the cells were incubated with primary antibody (HIF-1α) at 4 ^{o}C overnight. Subsequently, the cells were washed with PBS and incubated with secondary antibody with fluorophore for 30 min at room temperature. Finally, the cell nucleus was stained with DAPI. The cells were imaged by CLSM.

Starvation effect of 4T1 cells

4T1 cells were incubated in confocal dishes at 2×10^4 per dish overnight. Then the cells were incubated with a fresh medium (1 mL) containing GVs or TG-GVs (10μg mL^{-1}) for 5 h. Subsequently, a 10 μL of 2-NBDG solution (10 mM) was added into the dishes. After incubation for 30 min, the cell nucleus was stained with Hoechst 33342 before imaging by CLSM.

The calculation of combination index (CI):

According to the well-established Chou–Talalay method, combination index (CI) is determined as:

$$\mathrm{CI} = \frac{Dc_1}{D_1} + \frac{Dc_2}{D_2}$$

Where Dc_1 and Dc_2 are the doses of drug 1 and drug 2 in the combination therapy with a certain effect (e.g., 50% inhibition of cells) and D_1 and D_2 are the doses of the drugs at which the drugs have the same effect when administered singly. In this work, drug 1 was TPZ and drug 2 was GOx. For CI = 1, there is an additive effect; CI < 1, synergism; CI > 1, antagonism.

The calculation of Q value:

Q value method was used to analyze the synergistic interaction of combination efficiency of chemotherapy and starving therapy for tumor inhibition:

$$\mathrm{Q} = \frac{E_{(A+B)}}{(E_A + E_B - E_A \times E_B)}$$

Where $E_{(A+B)}$ represents the TGI of the TG-GV group. E_A and E_B represent the TGI of the monotherapy of T-GV and G-GV group, respectively. When $Q < 0.85$, there is an antagonism effect. When $0.85 \leq Q \leq 1.15$, there is an additive effect. When $1.15 < Q$, there is a synergistic interaction.

Supplementary Figures

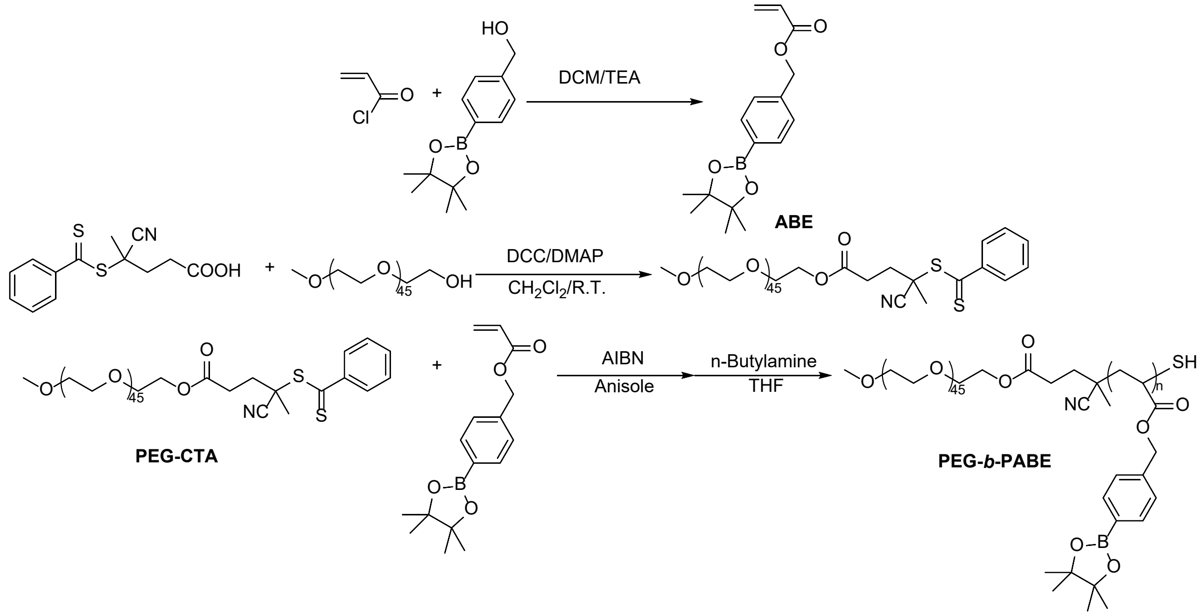

Figure S1. Scheme for the synthesis of PEG$_{45}$-*b*-PABE$_{330}$

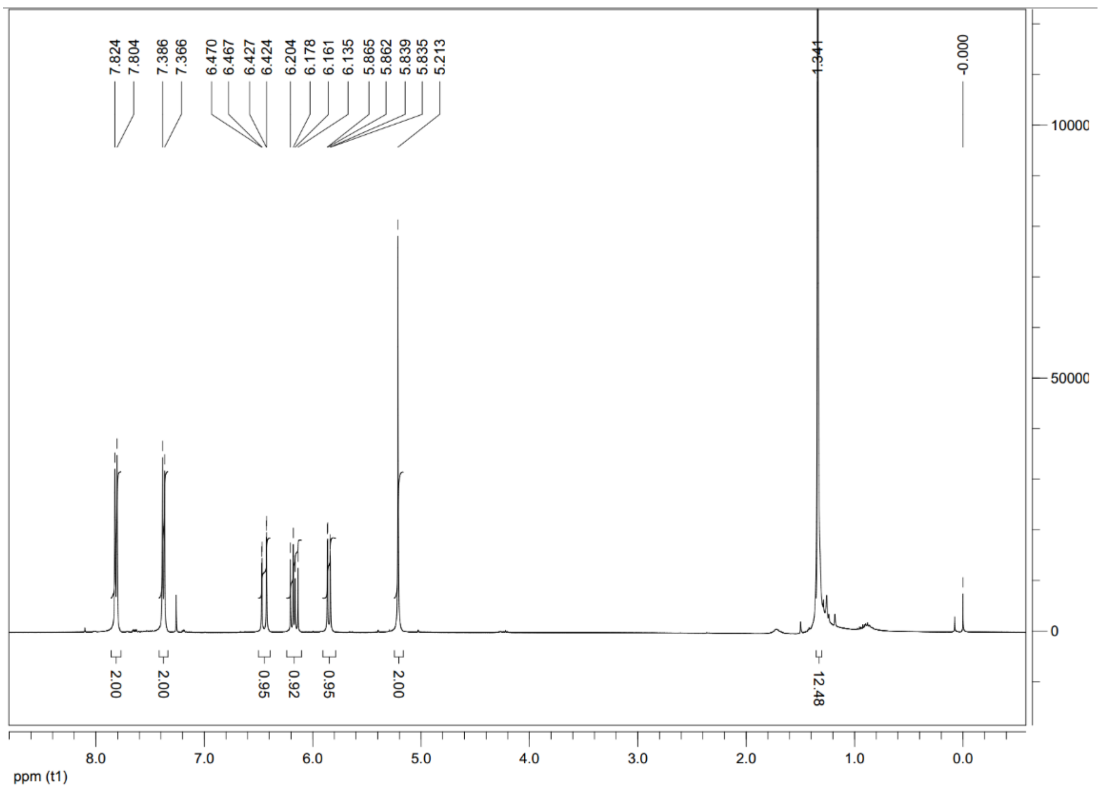

Figure S2. ^{1}H NMR of 4-(4,4,5,5-Tetramethyl-1,3,2-dioxaborolan-2-yl)benzyl acrylate (ABE).

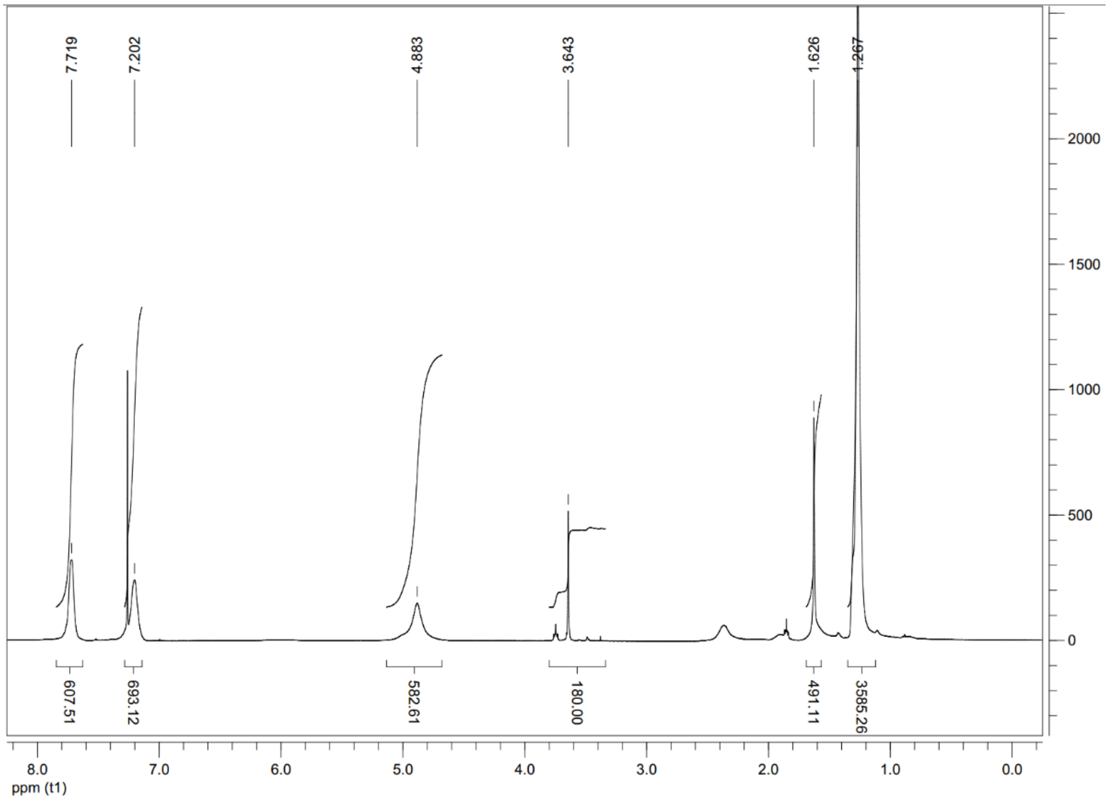

Figure S3. ^{1}H NMR of PEG$_{45}$-*b*-PABE$_{330}$ copolymer.

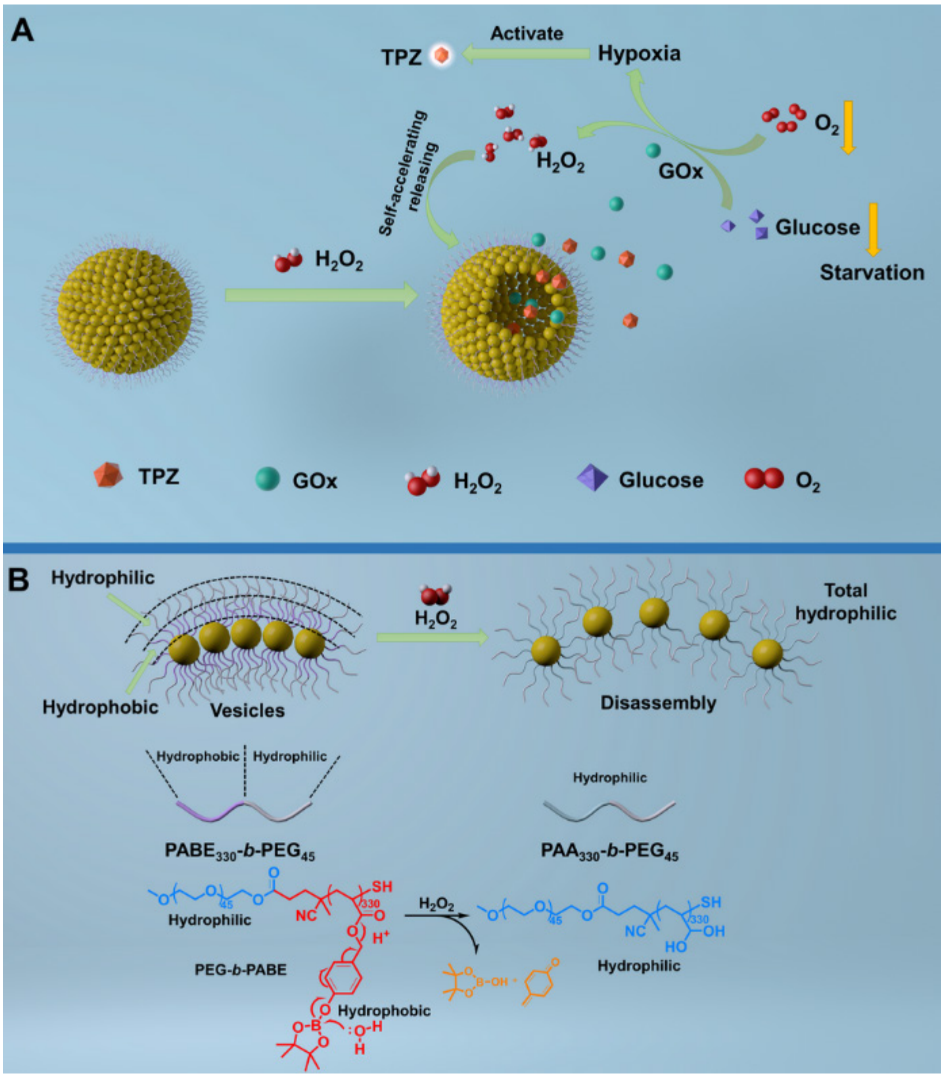

Figure S4. (A) Schematic illustration of the mechanism of controlled release of TPZ and GOx from TG-GVs in response to H_2O_2. (B) The structure change of the polymer chains after the oxidation by H_2O_2.

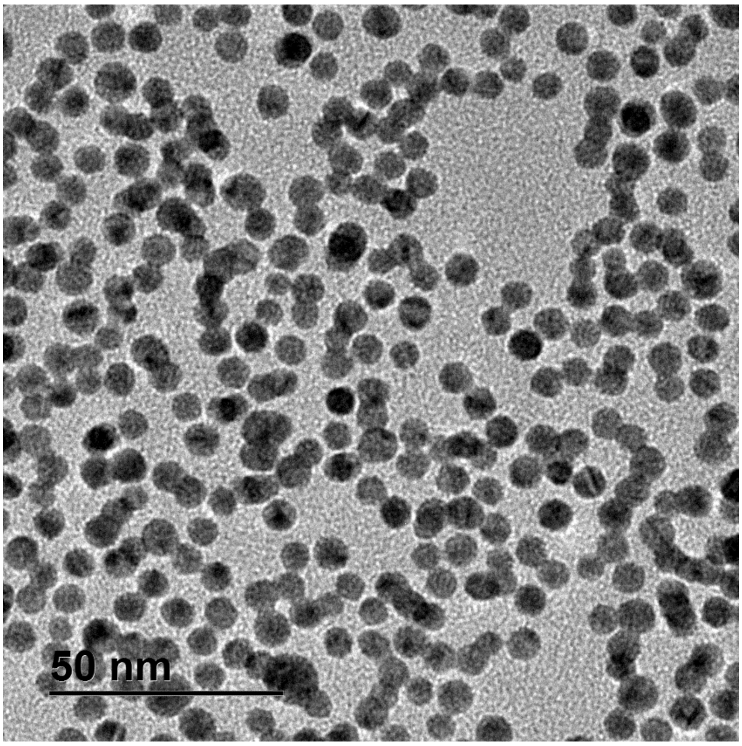

Figure S5. TEM image of GNPs with a dimeter of ~7 nm.

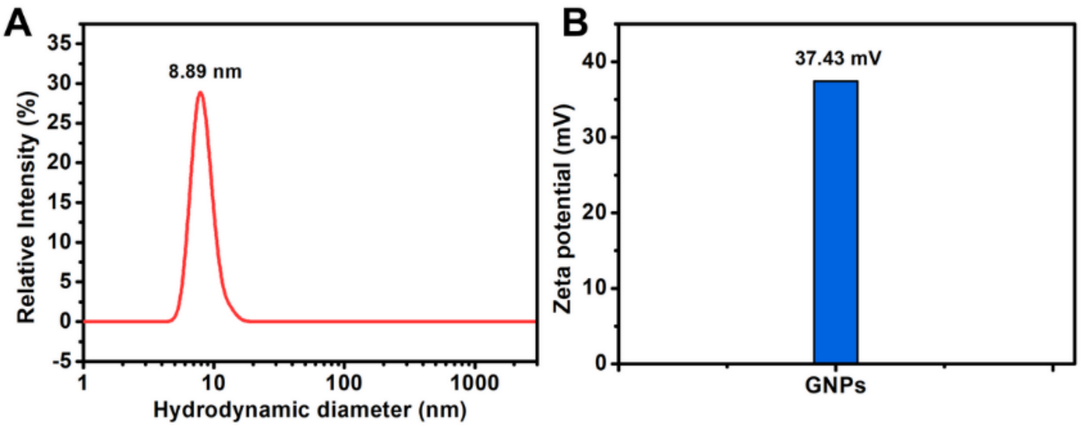

Figure S6. (A) DLS analysis of CTAB-covered GNPs. (B) Zeta potential of CTAB-covered GNPs.

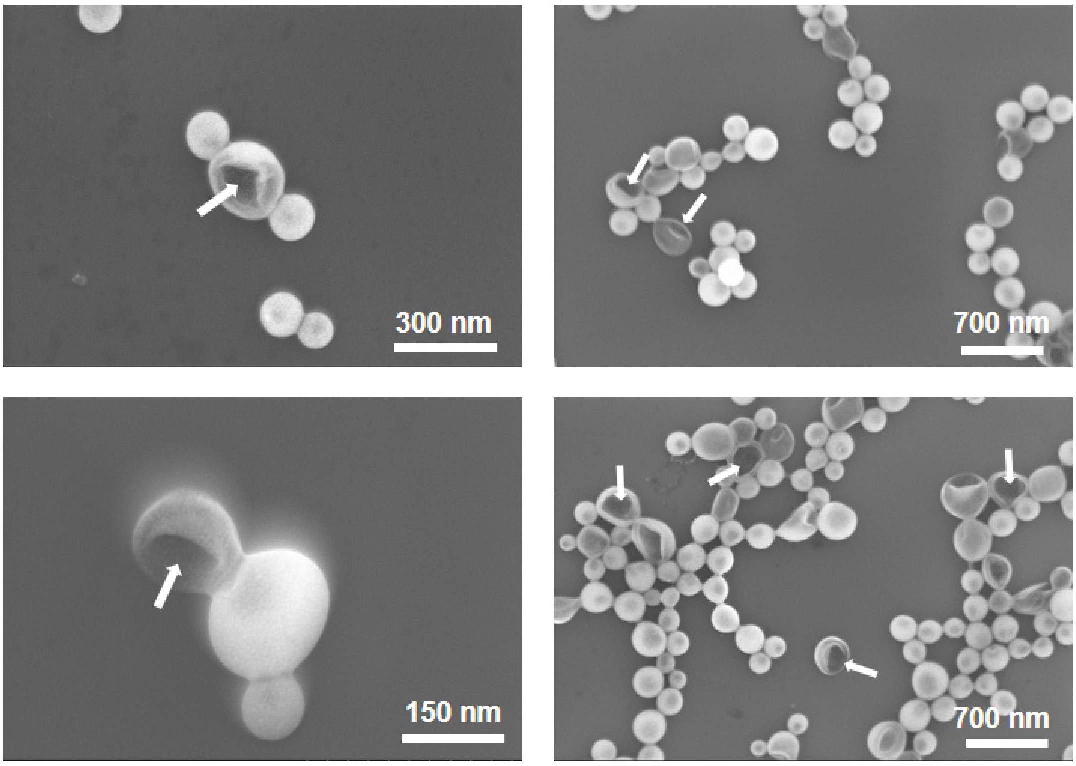

Figure S7. SEM images showing the cavity structure of TG-GVs. The arrows pointed to the cavity.

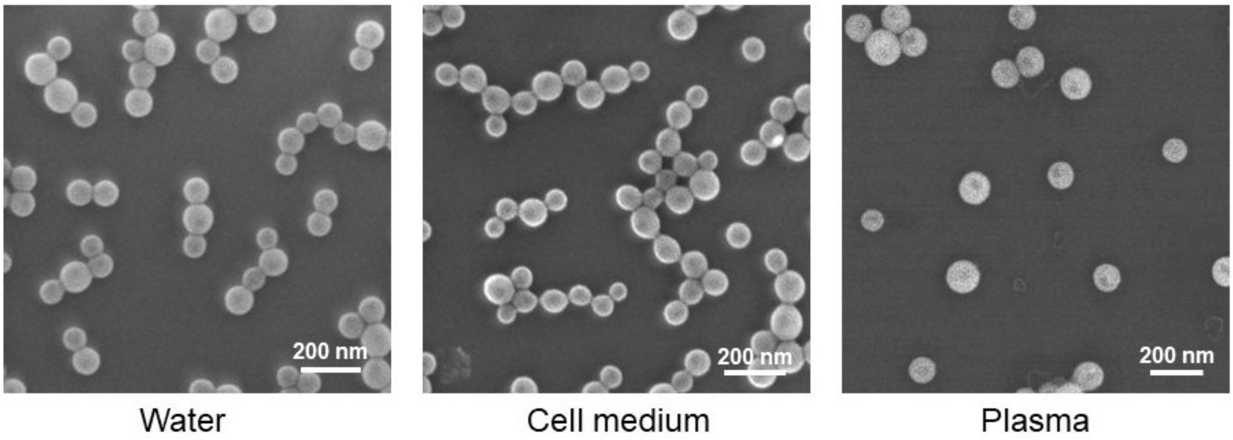

Figure S8. SEM images of TG-GVs after being incubated in water, cell medium and plasma of rats for 3 weeks.

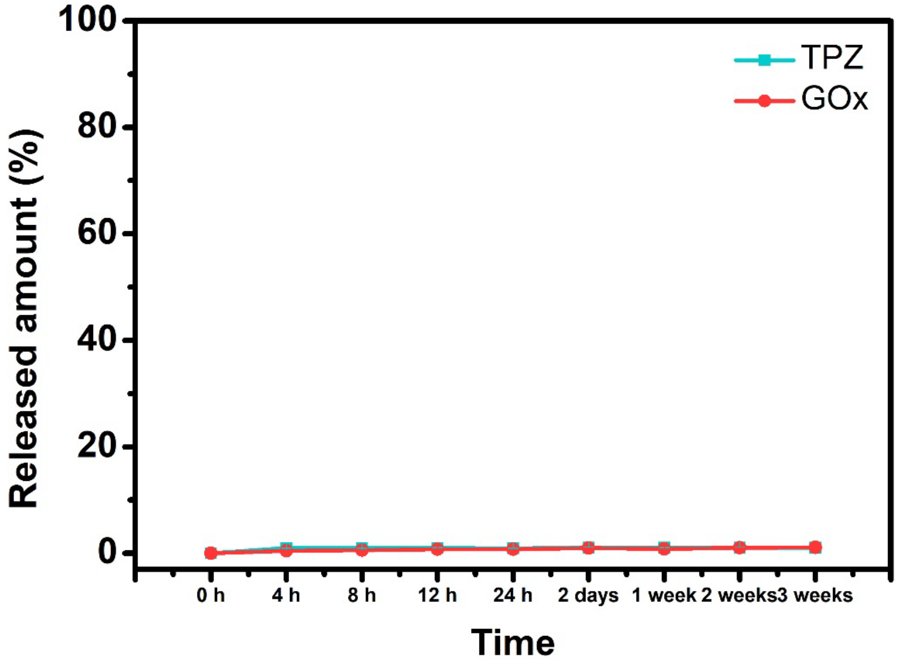

Figure S9. The release (or leakage) of TPZ and GOx from TG-GVs after the vesicles were dialyzed against water for 3 weeks.

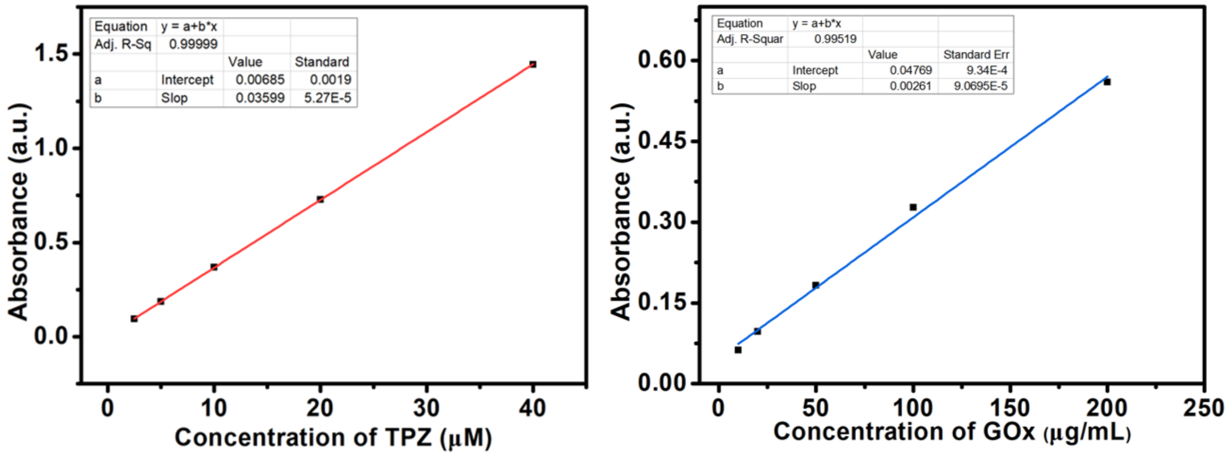

Figure S10. Standard curve of TPZ and BCA assay of GOx.

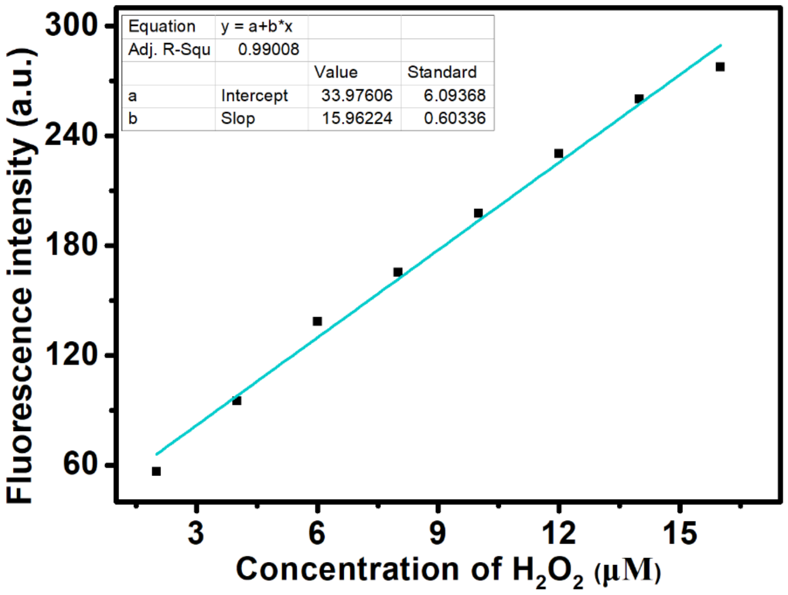

Figure S11. Standard calibration curve of the Amplex® Red assay of H_2O_2.

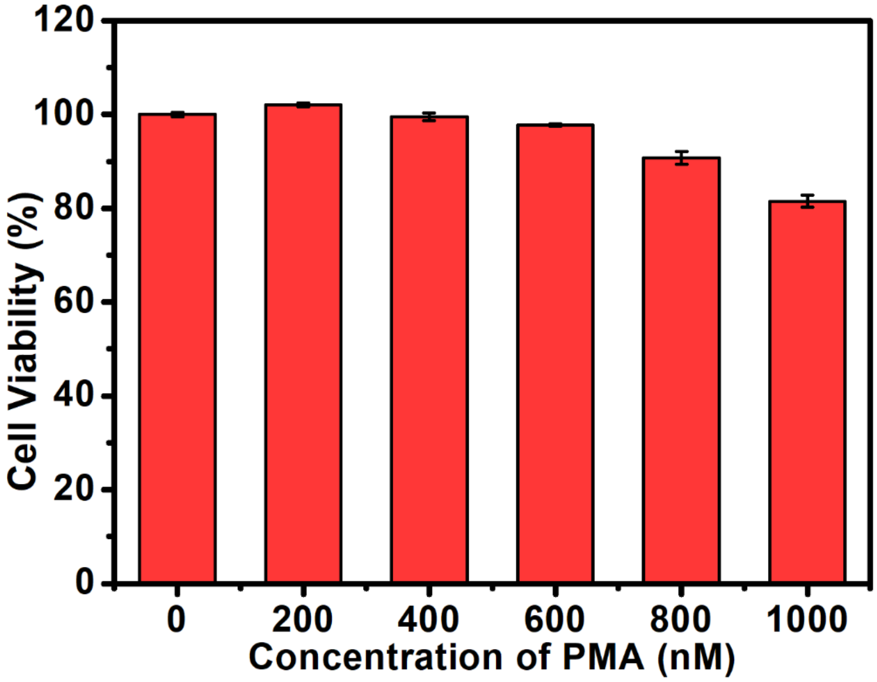

Figure S12. In vitro inhibition of 4T1 cells by PMA at different concentrations.

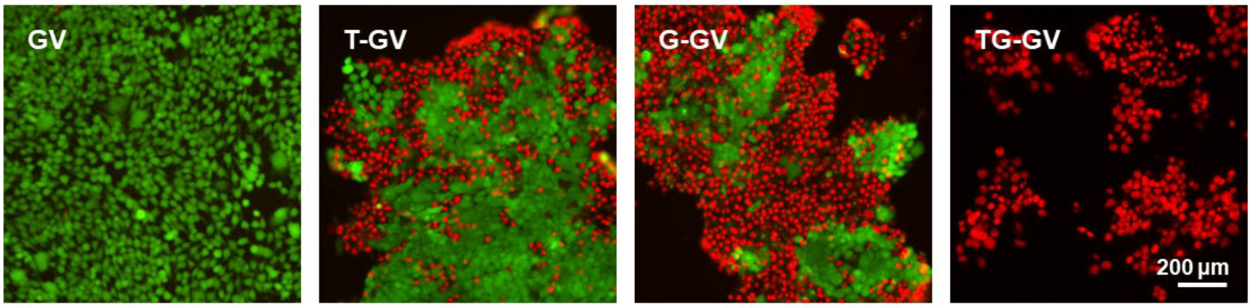

Figure S13. Live/dead assay of cells pre-treated with exogenous H_2O_2 (100 µM), followed by treating with GVs, T-GVs, G-GVs and TG-GVs. Live cells were stained with calcein AM (green), while dead cells were stained with propidium iodide (red). The scale bar is 200 µm.

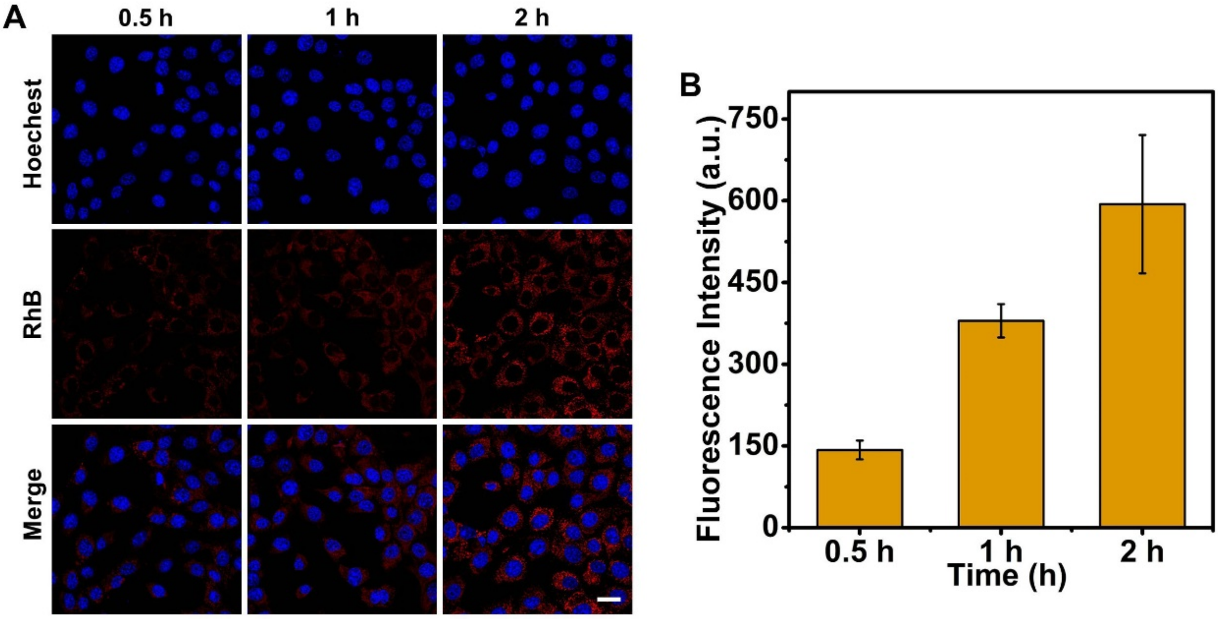

Figure S14. (A) CLSM observing of the endocytosis of TG-GVs by 4T1 cells at different time points. The scale bar is 25 μm. (B) Mean fluorescence intensity of the cells at different time points.

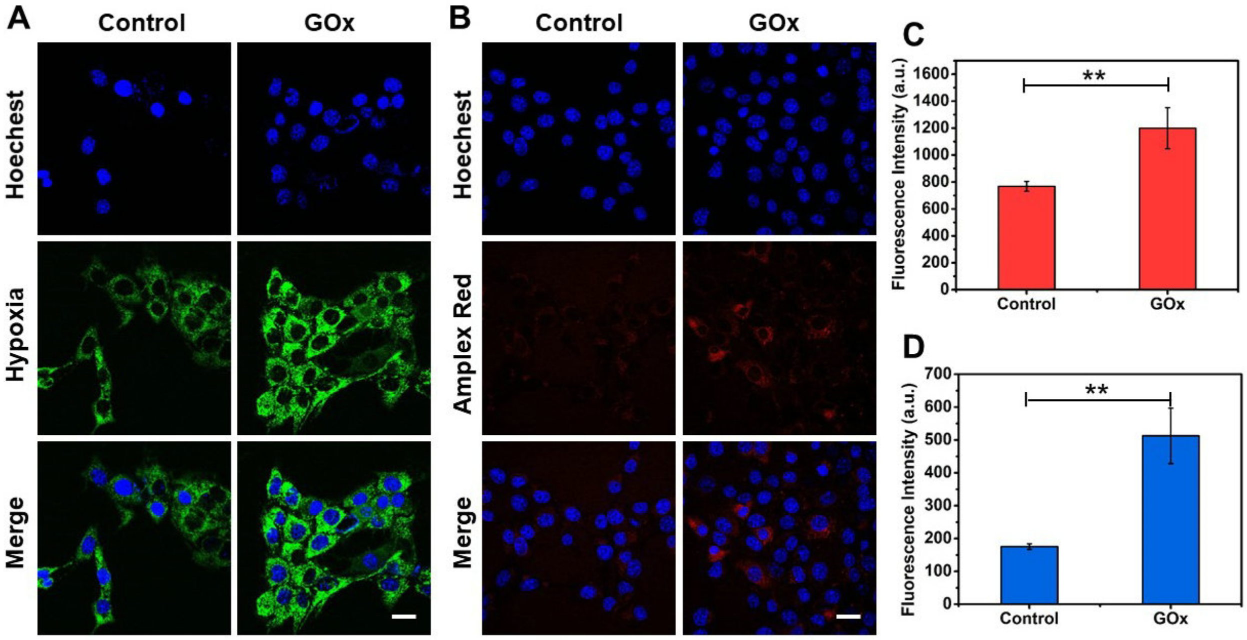

Figure S15. (A,B) CLSM observation of cellular hypoxia conditions (A) and H_2O_2 levels (B) of 4T1 cells treated with or without GOx. The scale bars are 25 μm. (C,D) Mean fluorescence intensity of the cells treated with or without GOx in hypoxia assay (C) and H_2O_2 assay (D). Data are the means ± s.d. ($n = 3$, $^{**}P < 0.01$).

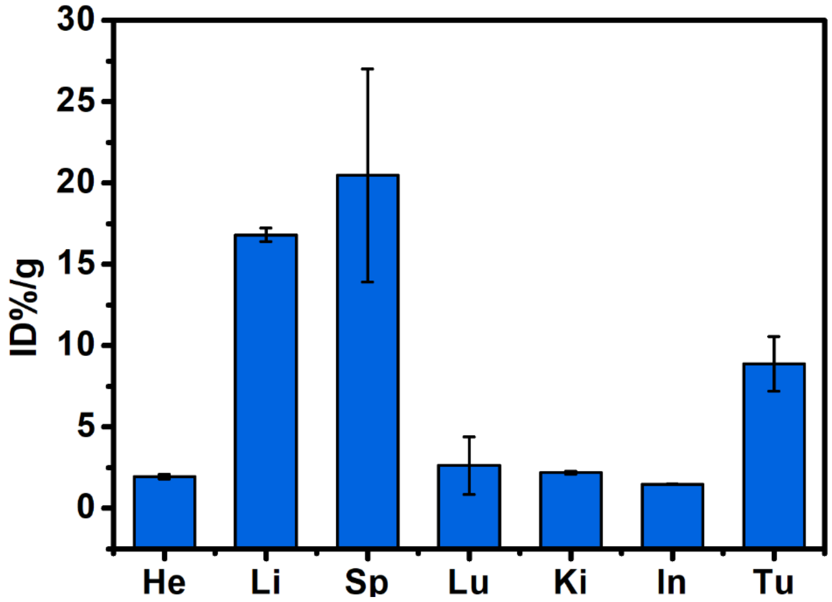

Figure S16. Quantitative ICP-OES analysis of TG-GVs in organs and tumors at 24 h post-injection. Data are the means ± s.d. (n = 3).

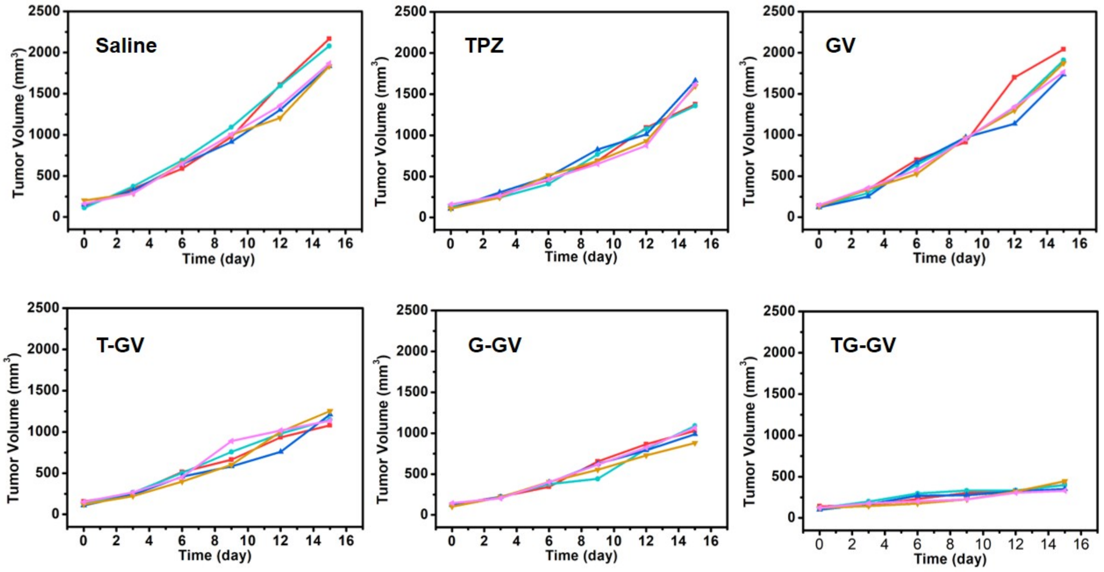

Figure S17. Tumor growth curves of mice treated by Saline, TPZ, GVs, T-GVs, G-GVs and TG-GVs.

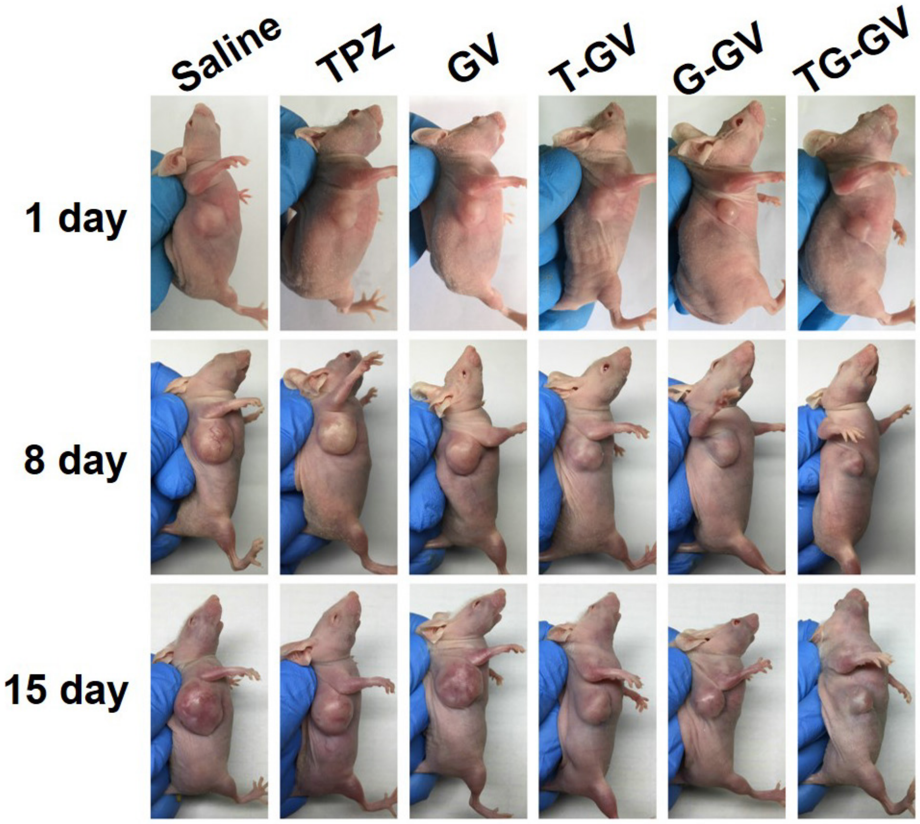

Figure S18. Representative photos of the mice under different treatments on day1, 8 and 15.

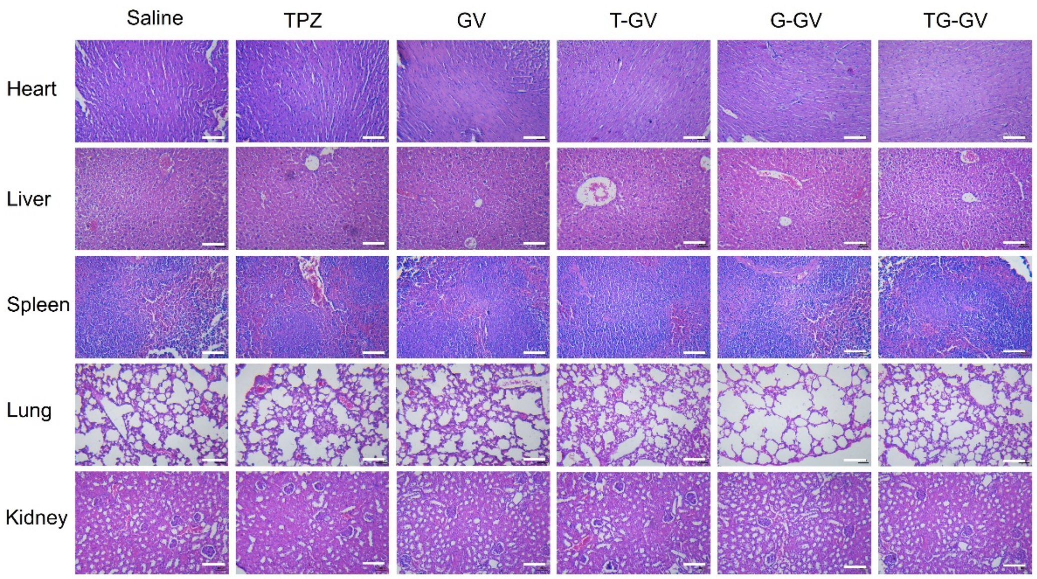

Figure S19. H&E staining of heart, liver, spleen, lung and kidney dissected from mice after the therapy. The scale bar is 200 μm.

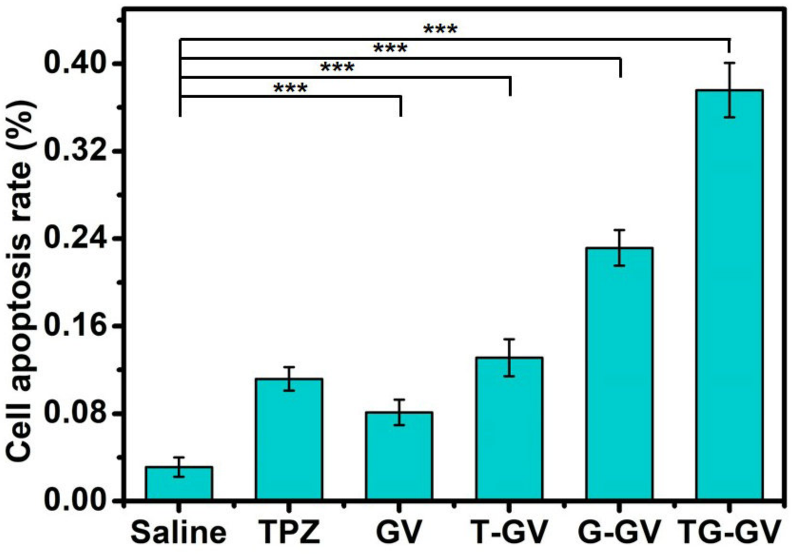

Figure S20. Apoptosis of cells treated with different groups of samples by TUNEL assay. Data are the means ± s.d. (n = 3, , ***P < 0.001).

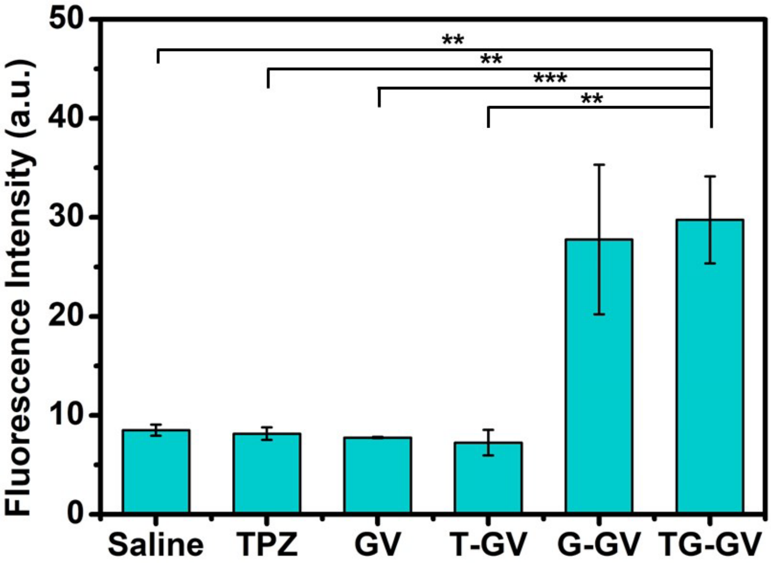

Figure S21. HIF-1α expression levels of cells treated with different groups of samples by HIF-1α immunofluorescence staining. Data are the means ± s.d. (n = 3, ***P < 0.001, **P < 0.01).
